# pH-responsive perylenediimide nanoparticles for cancer trimodality imaging and photothermal therapy

**DOI:** 10.7150/thno.36999

**Published:** 2020-01-01

**Authors:** Jianhao Li, Chang Liu, Yiseng Hu, Chendong Ji, Shuolin Li, Meizhen Yin

**Affiliations:** State Key Laboratory of Chemical Resource Engineering, Beijing Advanced Innovation Center for Soft Matter Science and Engineering, Beijing Laboratory of Biomedical Materials, Beijing University of Chemical Technology, No. 15 the North Third Ring Road East, Chaoyang District, Beijing 100029, PR China

**Keywords:** Perylenediimides, pH-responsive, Trimodality imaging, Phototheranostics

## Abstract

Organic chromophores have been well developed for multimodality imaging-guided photothermal therapy (PTT) due to their outstanding optical properties and excellent designability. However, the theranostic efficiencies of most currently available organic chromophores are restricted intrinsically, owing to their poor photostability or complex synthesis procedures. These drawbacks not only increase their cost of synthesis, but also cause side effects in PTT.

**Method**: We presented a facile strategy for constructing a near-infrared (NIR)-absorbing perylenediimide structured with pH-responsive piperazine ring at the bay region. The chromophore was conjugated with carboxyl-end-capped PEG as side chains that can self-assemble into nanoparticles (NPs) in aqueous solution. The NIR optical properties and photothermal conversation ability of PPDI-NPs were investigated. We then studied the imaging-guided PTT of PPDI-NPs under NIR light illumination in 4T1 cells and mice respectively.

**Results**: The excellent photostable PPDI-NPs had near-infrared fluorescence (NIRF) emission and high photothermal conversion efficiency in acidic microenvironment. Importantly, PPDI-NPs can be utilized for the precise detection of tumors by NIRF/photoacoustic/thermal trimodality imaging. Efficient PTT of PPDI-NPs was applied *in vitro* and *in vivo* with high biosafety.

**Conclusion**: In summary, we developed pH-responsive perylenediimide nanoparticles as multifunctional phototheranostic agent with high stability and simple synthesis procedures. This study offers a promising organic chromophore for developing phototheranostics in cancer therapy.

## Introduction

Cancer has been one of the main problems to human health. Currently, clinical cancer treatments have low therapeutic efficiency and unavoidable adverse effects 1. To tackle these challenges, nano-mediated therapy 2, especially photothermal therapy (PTT), has contributed to significant advances in this field 3-6. Photothermal transduction agents (PTAs) convert near-infrared (NIR) light (*λ* = 650-1100 nm) into heat, which can be exploited for tumor PTT 7,8. Compared with traditional treatments, such as chemotherapy and radiotherapy, PTT possesses minimal invasiveness and damage to normal tissues 9-11. However, without an accurate imaging guidance, PTT has unsatisfactory therapeutic effects. Hence, imaging-guided PTT has been developed as a promising therapeutic strategy for precise cancer therapy, as it can track the tumor lesion and evaluate the efficacy during the therapeutic process 12-14.

Various imaging modalities have been applied in cancer diagnosis. Among them, photoacoustic (PA) imaging has been considered as a promising noninvasive biomedical imaging modality. Based on optical excitation and ultrasound detection, PA imaging exhibits a high sensitivity of 100% and a penetration depth up to 7 cm 15, 16. Near-infrared fluorescence (NIRF) imaging is another modality with simple operation and easy data analysis for real-time monitoring of cancer treatments 17. NIRF imaging shows low background from autofluorescence and can be used to precisely identify tumor margins during tumor resection 18. Unfortunately, each of these two techniques has its own limitations, which make it difficult to obtain accurate and reliable diagnostic information individually 19, 20. For example, PA imaging is difficult to provide a whole-body imaging for small field of view 21, while fluorescence imaging is limited by poor tissue penetration 22. To overcome these intrinsic limitations, PTAs can be rationally designed to achieve multimodality imaging 23.

Organic chromophores have outstanding optical properties and excellent designability, which have been applied as new theranostic agents 24-26. Among them, cyanine-type chromophores, especially indocyanine green (ICG), have been widely investigated as multimodality image-guided PTAs 27. The inherent drawbacks of cyanine-type chromophores include poor photostability 28, short circulation time 29, and low tumor-to-background signal 30. Recently BODIPY-based PTAs have resolved some of these challenges, but raises other concerns such as complicated synthesis and purification 31. It is, therefore, desirable to develop stable and responsive organic chromophores with multimodality imaging functions for efficient cancer therapeutics.

**Scheme 1:**
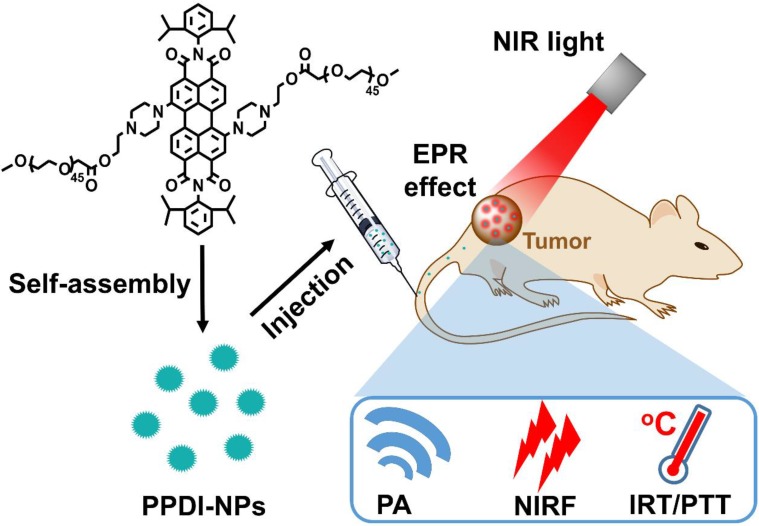
Schematic illustration of PPDI-NPs used for NIRF/PA/IRT imaging and photothermal therapy of tumor.

Herein, pH-responsive perylenediimide nanoparticles (PPDI-NPs) were developed for NIRF/PA/infrared thermal (IRT) trimodality imaging and PTT (Scheme 1). Previously, perylenediimides (PDIs) with high fluorescence intensity, chemical stability and biocompatibility 32 have been applied in cell imaging 33, DNA intercalator 34, drug/gene/protein delivery 35 and PA imaging-guided PTT 36, 37. In this study, a PDI molecule was structured with piperazine ring at the bay region, forming a typical push-pull structure with strong NIR absorption. The efficient photothermal conversion efficiency of PDI is beneficial for high contrast PA/IRT imaging and effective tumor photothermal ablation under irradiation (0.5 W/cm^2^). Importantly, the protonation of piperazines in acidic environment can block the photoinduced electron transfer (PET) process, resulting in an increase of NIRF emission (760 nm) 38-40. Thus, PPDI-NPs can serve as an effective NIRF agent for tumor imaging. Compared with previous theranostic agent, this stable and easily-manufactured perylenediimide nanoparticle contains the functionalities of trimodality imaging and PTT, which showed great potential in cancer theranostics.

## Experiment section

### Materials

1,7-Dibromo-3,4,9,10-perylenetetracarboxylic acid dianhydride (**PDI-2Br**, Hunan Hua Teng Pharmaceutical Co. Ltd., 95.55%), mPEG-COOH (2 KDa, Ponsure Biotechnology, 95%), dimethyl aminopyridine (DMAP), N-hydroxy benzotriazole (HOBT) and N, N-diisopropylcarbodiimide (DIC) (Alfa Aesar, 99%) were used without further purification. Deionized (DI) water (18.2 MΩ•cm resistivity at 25 ^o^C) was used for all tests. Cell counting kit (CCK-8), Calcein-AM and propidium iodide (PI) chromophore stuff were purchased from Dojindo Laboratories (Japan). Roswell park memorial institute-1640 was purchased from Solarbio. Other chemicals (Sigma Aldrich) were used without further purification.

### Synthesis of PPDI-NPs

The synthetic route of PDI (**4**) is shown in Scheme S1.

### 1,7-dibromo-N, N'-bis-(2,6-diisopropylphenly)-3,4:9,10-perylenediimide (2)

A mixture of compound **1** (1 g, 1.8 mmol), 2,6-diisopropylaniline (4.4 g, 24.8 mmol) and propionic acid (250 mL) was stirred for 48 hours at 140 ^o^C. After cooling to room temperature, the mixture was added into Na_2_CO_3_ solution (10%) (1.0 L) under stirring. Then the solid precipitate was washed with water, filtered and dried. The product was purified by silica gel column chromatography and preparative TLC (Solvent system: dichloromethane/n-hexane 70/30) to yield 1.1 g (75%) of yellow powder, compound** 2**. ^1^H NMR (400 MHz, CDCl_3_) δ 9.60 (d, J = 8.1 Hz, 2H), 9.02 (s, 2H), 8.82 (d, J = 8.2 Hz, 2H), 7.52 (s, 2H), 7.38 (d, J = 7.8 Hz, 4H), 2.72 (s, 4H), 1.20 (s, 24H).

### 1,7-bis-(4-(2-hydroxyethyl) piperazin-1-yl))-N, N'-bis-(2,6-diisopropylphenly)-3,4:9,10-perylenediimide (3)

A mixture of compound** 2** (1 g, 1.2 mmol), K_2_CO_3_ (1.55 g, 11.2 mmol) and N-(2-hydroxyethyl) piperazine (2.9 g, 22.3 mmol) in NMP (50 mL) was stirred for 48 hours at 85 ^o^C. After cooling to room temperature, the mixture was added into citric acid solution (5%) (1.0 L) under stirring. Then the solid precipitate was washed with water, filtered and dried. The product was purified by silica gel column chromatography and preparative TLC (Solvent system: dichloromethane/methanol 95/5) to yield 0.75 g (70%) of green powder compound **3**. ^1^H NMR (400 MHz, MeOD) δ 10.08 (s, 2H), 8.66 (s, 4H), 7.47 (s, 2H), 7.37 (s, 4H), 4.16 (s, 4H), 3.96 (s, 4H), 3.79 (s, 4H), 3.64 (s, 8H), 3.49 (s, 4H), 2.76 (d, J = 6.5 Hz, 4H), 1.15 (s, 24H). ^13^C NMR (101 MHz, MeOD) δ 165.53 (s), 151.35 (s), 147.61 (s), 142.90 (s), 137.61 (s), 132.26 (s), 131.99 (s), 131.28 (s), 130.87 (s), 127.31 (s), 125.30 (s), 124.31 (s), 123.00 (s), 73.32 (s), 71.59 (s), 65.25 (s), 57.01 (s), 30.60 (s), 24.50 (s). MS (ESI), m/z: Calcd. for: 967.2000, found: 967.5079 [M+H].

### 1,7-bis-(4-(2-PEGethyl) piperazin-1-yl))-N, N'-bis-(2,6-diisopropylphenly)- 3,4:9,10-perylenediimide (PPDI)

mPEG-COOH 2 KDa (500 mg, 0.25 mmol) was dissolved in anhydrous dichloromethane (10 mL). The solution was cooled to 0 ^o^C with stirring; this was followed by the addition of compound** 3** (120 mg, 0.1 mmol), DIC (158 mg, 1.3 mmol), HOBt (5.07 mg, 0.04 mmol), pyridine (100 mg, 1.3 mmol) under nitrogen. The temperature was maintained at 0 ^o^C for 2 h, then allowed to warm to room temperature with continued stirring overnight. After that, the crude product was dried to remove the solvent and purified by silica gel-based column chromatograph (Solvent system: dichloromethane/methanol 95/5). Removal of solvent and drying under vacuum afforded the purified green powder, compound** PPDI**. ^1^H NMR (400 MHz, CDCl_3_) δ 8.55 (s, 2H), 7.49 (s, 2H), 7.35 (d, 2H), 3.63 (s, 387H), 3.37 (s, 6H), 2.75 (s, 3H), 1.25 (s, 24H). MS (MALDI-TOF), m/z: Calcd. for: 5220.00, found 5251.62.

### Synthesis and characterization of PPDI-NPs micelles

Compound** PPDI** was dissolved in Deionized (DI) water to form PPDI-NPs micelles. Transmission electron microscopy (TEM, HT-7700, Hitachi, Japan) and Particle Size Analyzer (ZEN3600, Malvern) determined the size and morphology of PPDI-NPs. The UV-vis-NIR absorption spectrum of PPDI-NPs was recorded by spectrophotometer (UV-2450, SHIMADZU). The fluorescence spectrum of PPDI-NPs was recorded by (Horiba Jobin Yvon, NJ, USA). NIRF imaging was obtained by *In vivo* Imaging System (IVIS) Imaging Spectrum System and analyzed by an IVIS 3.0 Living Imaging software (PerkinElmer, U.S.). PA imaging of PPDI-NPs was measured via Multispectral Optoacoustic Tomography (MSOT) INVISIO-256 system (iThera Medical) using a phantom. The acidity constant (p*K*_a_) value of PPDI-NPs was calculated based on Henderson-Hasselbalch treatment according to the fluorometric results 41.


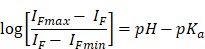


*I*_F_ corresponds to the fluorescent intensity of PPDI-NPs.

### Detection of singlet oxygen

The singlet oxygen generation of PPDI-NPs was tested following the Kraljic procedure 42. PPDI-NPs (100 μM) mixed with *p*-nitrosodimethylaniline (RNO, 50 μM), imidazole (50 μM), and PBS (20 mM, pH = 5.5 and 7.4) were exposed to laser (660 nm, 1 W/cm^2^) and monitored the absorbance at setting time. Water without PPDI-NPs was used as control. The singlet oxygen can blench the absorbance of RNO at 440 nm.

### Detection of photothermal effect

PPDI-NPs (200, 100, 50, 25, 12, 6 and 0 μM) in DI water were put into a cuvette and exposed to 660 nm laser (0.5 W/cm^2^) for 10 min respectively. To investigate the effect of laser power density, PPDI-NPs (100 μM) in DI water were put into a cuvette and exposed to 660 nm laser under different power intensity (0, 0.25, 0.5, 0.75 and 1.0 W/cm^2^) for 10 min respectively. To compare the PTT effect and stability, ICG (200, 100, 50, 25 and 0 μM) in DI water were put into a cuvette and exposed to 660 nm laser (0.5 W/cm^2^) for 10 min respectively. The IRT imaging and temperature data were recorded by a Fluke (Ti400) thermal imaging camera.

### Photothermal conversion efficiency test

PPDI-NPs (100 μM, pH 5.5 and 7.4) was put into a cuvette and exposed to 660 nm laser at 0.5 W/cm^2^. When the temperature reaches to maximum steady state, the PPDI-NPs cooled down naturally after the laser shut off. During the procedure, the temperature was recorded by thermal imaging camera. The photothermal conversion efficiency (*η*) was calculated according to reported work 43-45:


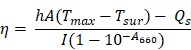


where *h* is the heat transfer coefficient, *A* is the surface area of the container, *T_max_* is the maximum steady-state temperature, *T_sur_* represents the ambient temperature of the environment, *Q_s_* is the heat dissipation of solvent (water) which was measured by a power meter (407A, Spectra-Physics), *I* is the incident laser power (0.5 W/cm^2^), and A_660_ is the absorbance of the PPDI-NPs at 660 nm. *hA* was calculated by the following equation:


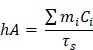


where *m* and *C* are the mass (1 g) and heat capacity (4.2 J/g) of water, respectively.

*τ*_s_ is the sample system time constant calculated by the following equation:


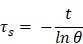


where *t* represents time. *θ* is the dimensionless driving force defined as (*T*-*T_sur_*)/(*T_max_*-*T_sur_*).

### Cell viability tested by CCK-8 assay

Mouse mammary carcinoma cell line 4T1 cells in the growth of log phase were cultured in 96-well plate at density of ~6000 cells/well and incubated for 24 h. Various concentrations of PPDI-NPs in culture media was added to each well (200 μL/well). After 24 h or 48 h exposed to PPDI-NPs, the culture media were changed back to fresh culture media, and cell viability was tested by Standard Cell Counting Kit-8 (CCK-8) assay.

### Photothermal therapy* in vitro*

4T1 cells in the growth of log phase were cultured in 96-well plate at density of ~6000 cells/well and incubated for 24 h. Various concentrations of PPDI-NPs in culture media was added to each well (200 μL/well) for 24 h incubation. The treated cells were exposed to laser irradiation (660 nm, 0.5 W/cm^2^, 10 min). Cell viability after PTT was investigated by Standard Cell Counting Kit-8 (CCK-8) assay. After photothermal treatment, the four groups, cells with PBS, only PPDI-NPs, only laser and PPDI-NPs with laser, were co-stained with Calcein-AM and PI, and monitored by EVOS® FL Auto Cell Imaging System.

### Animal model

Female BALB/c mice (6-week) were obtained from Beijing Huafukang Biological Technology Co., Ltd. (China). To construct the tumor model, mice were anaesthetized and subcutaneous injected 50 μL of 4T1 cells (1 × 10^6^) suspension. Tumor volume (*V*) was computed as *V* = width^2^ × length/2. Ten days later, the tumor imaging and therapy studies were carried out when the tumor size reached about 125 mm^3^ (width = 6 mm, length = 7 mm). All animal care and experimental protocols complied with the Animal Management Rule of the Ministry of Health, People's Republic of China (Documentation no. 55, 2001) and the Guide for the Care and Use of Laboratory Animals published by the US National Institutes of Health (NIH Publication no. 85-23, revised 1996).

### *In vivo* NIRF/PA imaging

100 μL of PPDI-NPs (3.47 mg/kg) was intravenously injected into the tumor-bearing nude mice. *In vivo* NIRF imaging were acquired at predetermined time points by IVIS Imaging Spectrum System and analyzed by an IVIS system under certain parameters (*λ*_ex_ = 640 nm and *λ*_em_ = 650-800 nm, exposure time = 0.1 s). At the same time, the administered mice were imaged using the MSOT INVISIO-256 system (iThera Medical). The excitation wavelength was set from 680 to 800 nm with a 10 nm interval. At the post-injection time of 24 and 48 h, the mice were sacrificed. Organs including heart, liver, spleen, lung, kidney, muscle and tumors were harvested for the *ex vivo* imaging and semiquantitative biodistribution analysis.

### *In vivo* IRT imaging and PTT

Mice bearing 4T1 tumor were divided into four groups for various treatment: PBS injection only, PPDI-NPs injection only, and laser irradiation only, PPDI-NPs injection and laser irradiation. 100 μL of PPDI-NPs (3.47 mg/kg) was intravenously injected into the mice. An equal volume of PBS buffer solution was injected as control. After 24 h post-injection, the tumor site of the mice of experiment and control group were exposed to 660 nm laser irradiation at 0.5 W/cm^2^ for 10 min. During the experiment, the temperature of tumor was recorded by thermal imaging camera. The body weight and tumor size were recorded every two-day at post-treatment. After the end of the experiment (22 d), the mice were sacrificed, and tumor tissue was dissected for H&E staining.

### *In vivo* toxicity assessment of PPDI-NPs

100 μL of PPDI-NPs (3.47 mg/kg) was intravenously injected into the healthy BALB/c mice (*n* = 3). At the 15 days and 30 days, blood samples (2.0 mL) were collected by removal of the mice eyeballs and centrifuged twice at 3000 rpm for 10 min to collected serum for biochemical assays. The major organs (heart, liver, spleen, lung and kidney) were collected for hematoxylin & eosin (H&E) staining.

### Semi-quantitative pharmacokinetic studies

100 μL of PPDI-NPs (3.47 mg/kg) was intravenously injected into different groups of healthy BALB/c mice (*n* = 3). About 10-20 μL blood samples were drawn from the tail vein at certain time point. Next, blood samples were solubilized with lysis buffer (RIPA) and measured by a fluorometer to determine the PPDI-NPs concentration (*λ_ex_* = 660 nm, *λ_em_* = 760 nm). The fluorescence intensity of PPDI-NPs was calculated by deducting the blank control blood sample. The PPDI-NPs level in the blood was presented as the percentage of injected dose per gram tissue (% ID g^-1^).

### Statistical Analysis

Date are reported as mean ± SD. Normally distributed data were analyzed using parametric testing. Unpaired Student's t-tests was for two groups. In all tests, statistical significance was set at *p* < 0.05 (*).

## Result and Discussion

### Characterization and NIR optical properties of PPDI-NPs

The pH-responsive perylenediimide was facilely synthesized by a coupling reaction between bromine-substituted PDIs and piperazine ring. The detailed syntheses route was described in Scheme S1. First, commercially available **PDA-2Br** (**1**) was converted to the corresponding perylenediimides (**2**) using 2,6-diisopropylaniline. The compound **2** was reacted with N-(2-hydroxyethyl) piperazine in dry NMP. The resultant compound was purified to obtain compound **3** as green powder. Compared with compound **2**, compound **3** has a maximum absorbance in the NIR range and large Stokes shift (>140 nm), as shown in Figure S5A. The introduction of amine, an electron donor, to PDI scaffold can red shift the excitation and emission wavelength of PDI through push-pull effect, but quench the fluorescence significantly (Figure S5B). Finally, a carbodiimide-mediated esterification was employed to conjugate carboxyl-end-capped PEG as side chains as the final product, compound **PPDI**. The structures of the intermediate and final product were confirmed by ^1^H NMR (Figure S1, S2, S6), ^13^C NMR (Figure S3), and MS spectra (Figure S4, S7), as shown in the Supporting information.

**Figure 1:**
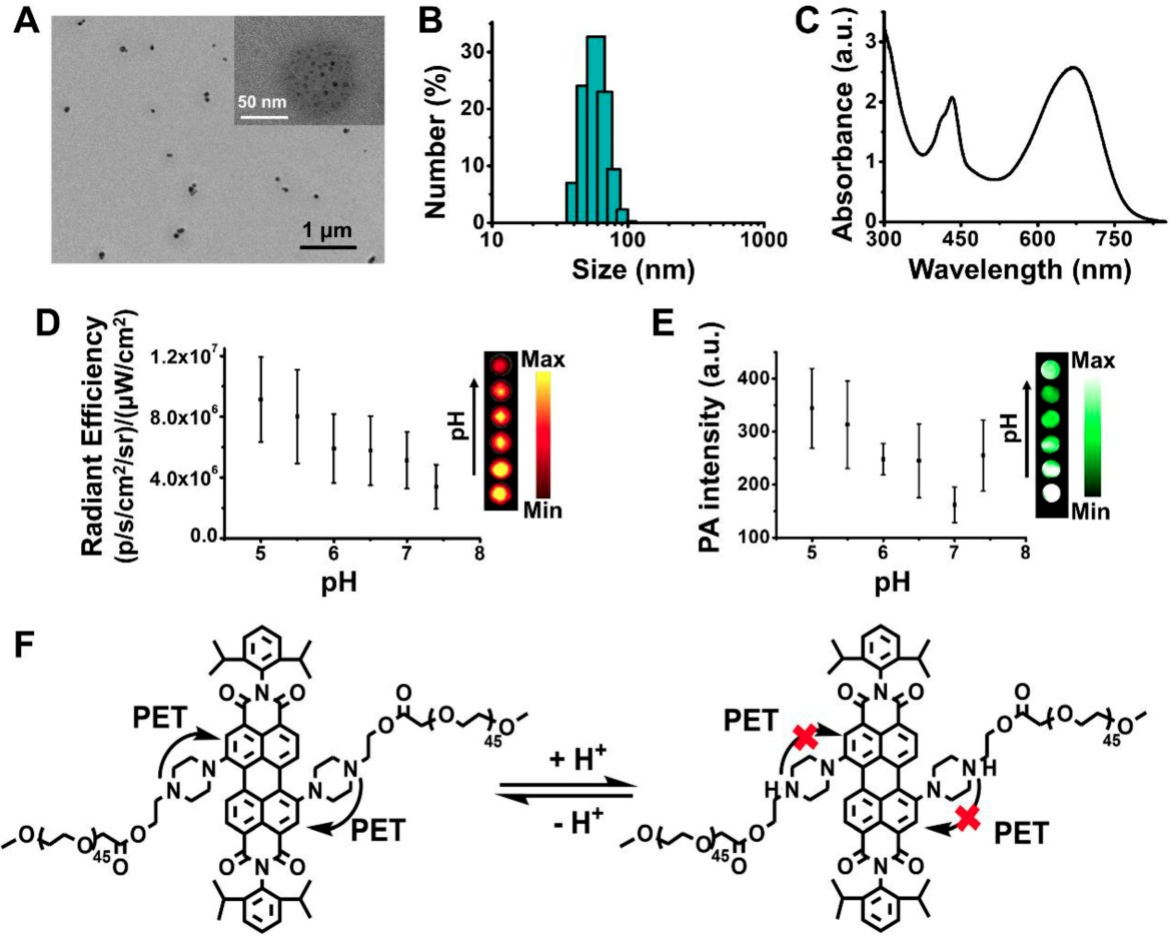
Characterization of PPDI-NPs. (A) TEM images. (B) DLS. (C) Absorbance spectrum. (D) NIRF imaging and quantification of PPDI-NPs (100 μM) at different pH. (E) Multispectral optoacoustic tomography system (MSOT) imaging and quantification of PPDI-NPs (100 μM) at different pH using a phantom. (F) pH-response of PDI core based on PET mechanism which can be blocked by the protonation of piperazines ring.

According to its structure, compound** PPDI** is amphiphilic with PDI core and hydrophilic PEG segment, which can self-assemble into nanoparticles (PPDI-NPs) in water. Transmission electron microscopy (TEM) images revealed that PPDI-NPs had uniform morphology with a diameter of approximately 60 nm (Figure 1A). The black dots possessing high contrast in TEM image in the PPDI-NPs were small aggregates of PDI core, which is due to high electron cloud density of benzene rings in PDI 46-48. The TEM images of PPDI-NPs at different pH were shown in Figure S8D. The size of PPDI-NPs slightly decreased with the decrease of pH. The pronation of the piperazine ring can result in the deaggregation of the PDI core. Dynamic light scattering (DLS) data showed the average hydrodynamic size of the nanoparticle is 65.2 ± 1.4 nm with a narrow polydispersity index (pdi) of 0.28 (Figure 1B). At lower pH, the size of PPDI-NPs decreased and the size at pH = 5.5 is 50.7 nm (pdi = 0.35, Figure S8A), which is benefit for deep penetration into tumor tissues. The DLS data of PPDI-NPs at pH 7.4 or 5.5 remained almost the same after storage in PBS for several days (Figure S8B). Zeta potential of PPDI-NPs in various pH solutions were shown in Figure S8C. PPDI-NPs had weak negative zeta potential at pH 3~7.4, owing to non-charge PEG shell. The zeta potential of PPDI-NPs increased to +7.74 mV when the piperazine was fully protonated at pH = 1, while decreased to -11.5 mV at pH = 11. The protonation and deprotonation of piperazine ring resulted in zeta potential changes of PPDI-NPs 40. Therefore, the well-defined molecular structure of compound **PPDI** provided PPDI-NPs with excellent stability.

Notably, the as-prepared PPDI-NPs aqueous solution exhibited strong absorption in the spectral region of 600-760 nm with a maximum absorption at 670 nm (Figure 1C), owing to the pull-push structure. Usually, it is inevitable that electron-donating groups in perylene's bay-region cause the fluorescence quenching through the intramolecular charge transfer (ICT) and PET effect. In our work, the PET from methyl-substituted nitrogen atoms to the perylene chromophore was found to be blocked in acid environment. As expected, varying the pH from basic (11) to acid (3), the fluorescence of PPDI-NPs solution was significantly enhanced and slightly blue shifted (Figure S9A-B**)**. The fluorescence quantum yield (Ø_f_) was measured at room temperature using Zinc phthalocyanine in toluene (Ø_f_ = 0.30) as standard 49. The Ø_f_ of PPDI-NPs in acidic microenvironment (pH = 5.5) was calculated as 0.02. Based on our results, the absorption of PPDI-NPs slightly changed along with the change of pH (the *p*K_a_ of PPDI-NPs can be determined as 5.2), which agrees with a previously reported work (Figure S9C) 40. Observations with an *In vivo* Imaging System (IVIS) proved a linear relationship between NIR fluorescence and concentration of PPDI-NPs in PBS, as shown in Figure S10A. The NIRF intensity at pH 5.0-7.4 was investigated by IVIS system. As shown in the Figure 1D, the NIRF intensity at pH 5.0 is stronger that that at pH 7.4, showing a pH-responsive behavior. The *in vitro* PA imaging of PPDI-NPs in a water bath was evaluated on a MSOT system. The photoacoustic signal was linearly increased with the increase of PPDI-NPs concentration (Figure S10B), highlighting the potential of PPDI-NPs for PA imaging. Importantly, amplified PA signal were shown at the lower pH (Figure 1E), which is suitable for applications under acidic tumor environment. So far, most of the reported NIR-absorbing perylenediimide had very weak fluorescence emission, which was not suitable for NIRF imaging. In this work, owing to the pH-responsive capacity (Figure 1F), PPDI-NPs have both NIRF and PA properties, which is suitable for multimodality imaging. These finding indicated the potential of PPDI-NPs unprecedented advantages of NIRF/PA imaging ability.

### Photothermal effect of PPDI-NPs

**Figure 2:**
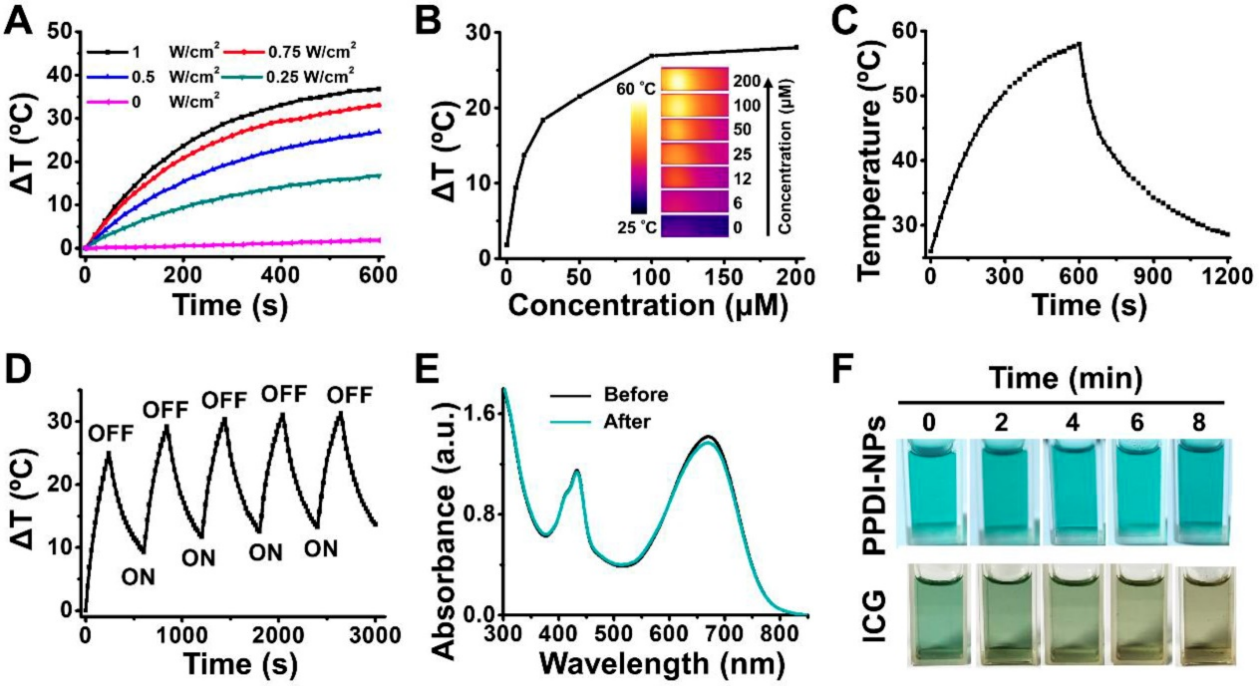
Photothermal properties of PPDI-NPs. (A) Laser power-dependent temperature change (ΔT) of PPDI-NPs (100 μM) under the power density of 0, 0.25, 0.5, 0.75, 1.0 W/cm^2^. (B) Relationship between PPDI-NPs concentration and temperature change. The insert pictures are the IRT imaging in test (0.5 W/cm^2^, 10 min). (C) Photothermal effect of PPDI-NPs (100 μM) in DI water exposed to laser (0.5 W/cm^2^). The laser was turned off after 10 min. (D) Photothermal stability test of PPDI-NPs (100 μM) in DI water exposed to laser (1.0 W/cm^2^). (E) Absorption spectrum of PPDI-NPs (100 μM) before and after laser irradiation (1.0 W/cm^2^, 10 min). (F) Photographs of PPDI-NPs (100 μM) and ICG (100 μM) at different irradiation times (1.0 W/cm^2^, 10 min). A 660 nm laser was used in the test.

Another important attribute of PPDI-NPs is their photothermal effect. To evaluate the photothermal properties, PPDI-NPs were dissolved in aqueous solution and exposed to a 660 nm NIR laser with different laser power densities. The temperature was monitored with a thermal imaging camera. As shown in Figure 2A, the temperature of PPDI-NPs solution increased with the increase of laser power. To enhance therapeutic efficiency and minimize damage to normal tissue, a mild irradiation intensity (0.5 W/cm^2^) was chosen for following experiments. Under this irradiation, the temperature of PPDI-NPs solution rapidly increased, depended on the PPDI-NPs concentration (Figure S11A and Figure 2B). The temperature of solution was raised by 53 °C when the PPDI-NPs' concentration was 100 μM at 0.5 W/cm^2^ irritation, while DI water increased only ~3 °C under the same irradiation condition. The strong photothermal conversion properties of PPDI-NPs paved the way for IRT imaging, in which the intensity of signal reflects the concentration as seen in Figure 2B. The IRT imaging has advantages including agent location identifying and process monitoring during the PTT 50, 51. To assess the photothermal conversion efficiency, a PPDI-NPs solution (100 μM) was exposed to assess a laser (660 nm, 0.5 W/cm^2^) and allowed to reach a steady-state temperature before being cooled down naturally, as showed in Figure 2C. Based on the data (Figure S11B), the photothermal conversion efficiency (*η*) was calculated to be 45.3% in DI water, which was similar to the reported perylenediimide-based PTAs 36, 48, 52, 53.

Photothermal stability is an important factor for PTAs. To investigate the photothermal stability, a PPDI-NPs solution (100 μM) was irradiated by a laser with relative high-power intensity (1 W/cm^2^) for five heat-cool cycles. As shown in Figure 2D, the temperature of PPDI-NPs increased when the irradiation was on and decreased when the irradiation was off during each ON/OFF cycle, showing an excellent photothermal stability. The ΔT in later cycles was about 5 °C higher than the first cycle because the solution did not completely cool back to room temperature before each new heating cycle 44, 45. Moreover, there is no obvious change of the absorbance spectrum (Figure 2E), morphology (Figure S12A) and size (Figure S12B) before and after laser irradiation, indicating the good photostability of PPDI-NPs. In sharp contrast, ICG solution showed remarkably absorption reduction and obvious change of appearance under the same experiment condition (Figure S13A and Figure 2F). PPDI-NPs also showed more significant temperature change than ICG under the same condition (Figure S13B-C). Considering the pH-dependent response of PPDI-NPs, PTT in acidic microenvironment (pH = 5.5) was investigated. As shown in Figure S14A-B, the photothermal conversion ability of PPDI-NPs decreased with pH decrease. The calculated *η* slightly decreased from 45.3% (pH 7.4) to 40.3% (pH 5.5). As reported, amine (N) substitution at the bay region of PDI leads to enhanced photo-to-heat conversion ability via push-pull effect 48. Thus, at pH 5.5, partial protonation of N to NH+ results in decreased photo-to-heat conversion and enhanced fluorescence. Low reactive oxygen species generation rate of PPDI-NPs (pH = 5.5 and 7.4) were detected under laser irradiation (Figure S15), indicating that PPDI-NP has no obvious photodynamic effect. Thus, vibrational relaxation may play major role in energy conversion and photothermal effect of PPDI-NPs 54, 55. All the results indicated that PPDI-NPs can be applied as an excellent PTA for IRT imaging and provide real-time monitoring of temperature dynamics during PTT process.

### Cellular investigation of PPDI-NPs

**Figure 3:**
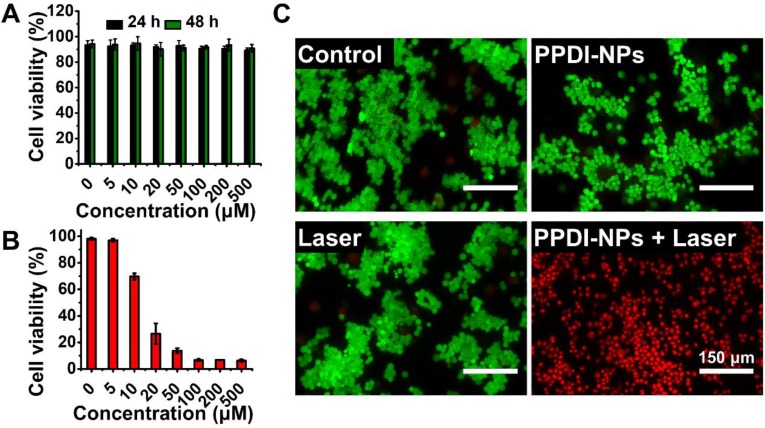
Viability of 4T1 cells by CCK-8 assay. (A) After cultured with various concentrations of PPDI-NPs for 24 h and 48 h. (B) After cultured with various concentrations of PPDI-NPs and exposed to laser (660 nm, 0.5 W/cm^2^, 10 min). (C) Fluorescence imaging of Calcein AM (green, live cells) and Propidium iodide (red, dead cells) co-stained 4T1 cells treated with PBS, PPDI-NPs only, laser only and PPDI-NPs with laser. Scale bars = 150 μm.

The biocompatibility of PPDI-NPs was investigated using a CCK-8 assay with the mouse mammary carcinoma cell line 4T1. As seen from Figure 3A, the cell viability was approximately 90% even cultured with high concentration PPDI-NPs (500 μM) for a long period of time (48 h), indicating the good biocompatibility and low toxicity of PPDI-NPs *in vitro*. Encouraged by the excellent photothermal effect of PPDI-NPs, we further investigated *in vitro* PTT under laser irradiation (0.5 W/cm^2^). As depicted in Figure 3B, with 660 nm laser irradiation, PPDI-NPs showed a dose-dependent cytotoxicity against 4T1 cells, indicating efficient PTT *in vitro*. To visually evaluate this therapeutic effect, live cells and dead cells were identified by co-staining Calcein-AM (green fluorescence; living cells) and Propidium iodide (red fluorescence; dead cells) after laser treatment. As shown in Figure 3C, all the cells displayed green fluorescence in control groups, which suggests that pure laser or PPDI-NPs failed to kill the 4T1 cells. In contrast, the cells displayed red fluorescence when they were incubated with PPDI-NPs (100 μM) and exposed to a 660 nm laser (0.5 W/cm^2^, 10 min), suggesting that PPDI-NPs generated enough heat by laser irradiation to kill the 4T1 cells. The *in vitro* studies proved that PPDI-NPs could sever as potential biocompatible PTAs for photothermal ablation of cancer cells.

### *In vivo* NIRF/PA imaging of PPDI-NPs

Combining different advantages of each imaging, multimodality molecular imaging provides precise detection of PATs to avoid damages to surrounding benign tissues during PTT. For *in vivo* animal studies, BALB/c mice were intravenously (i.v.) injected with PPDI-NPs when the tumor size reached about 125 mm^3^.

By measuring the fluorescence intensity of PDI at 760 nm, the post-injection concentration of PDI was calculated to determine the pharmacokinetics profile of PPDI-NPs. As shown in Figure S16, blood circulation of PPDI-NPs showed a typical two compartment model: the first phase (distribution phase) with a half-life of only 2.14 ± 0.23 h, and the second phase (elimination phase, the process for NPs clearance) with a half-life of 22.23 ± 3.38 h. PPDI-NPs exhibited a long blood circulation in the second phase (circulation phase), owing to the PEG coating of nanoparticles that delayed their macrophage clearance in reticuloendothelial systems (RES) 56.

**Figure 4:**
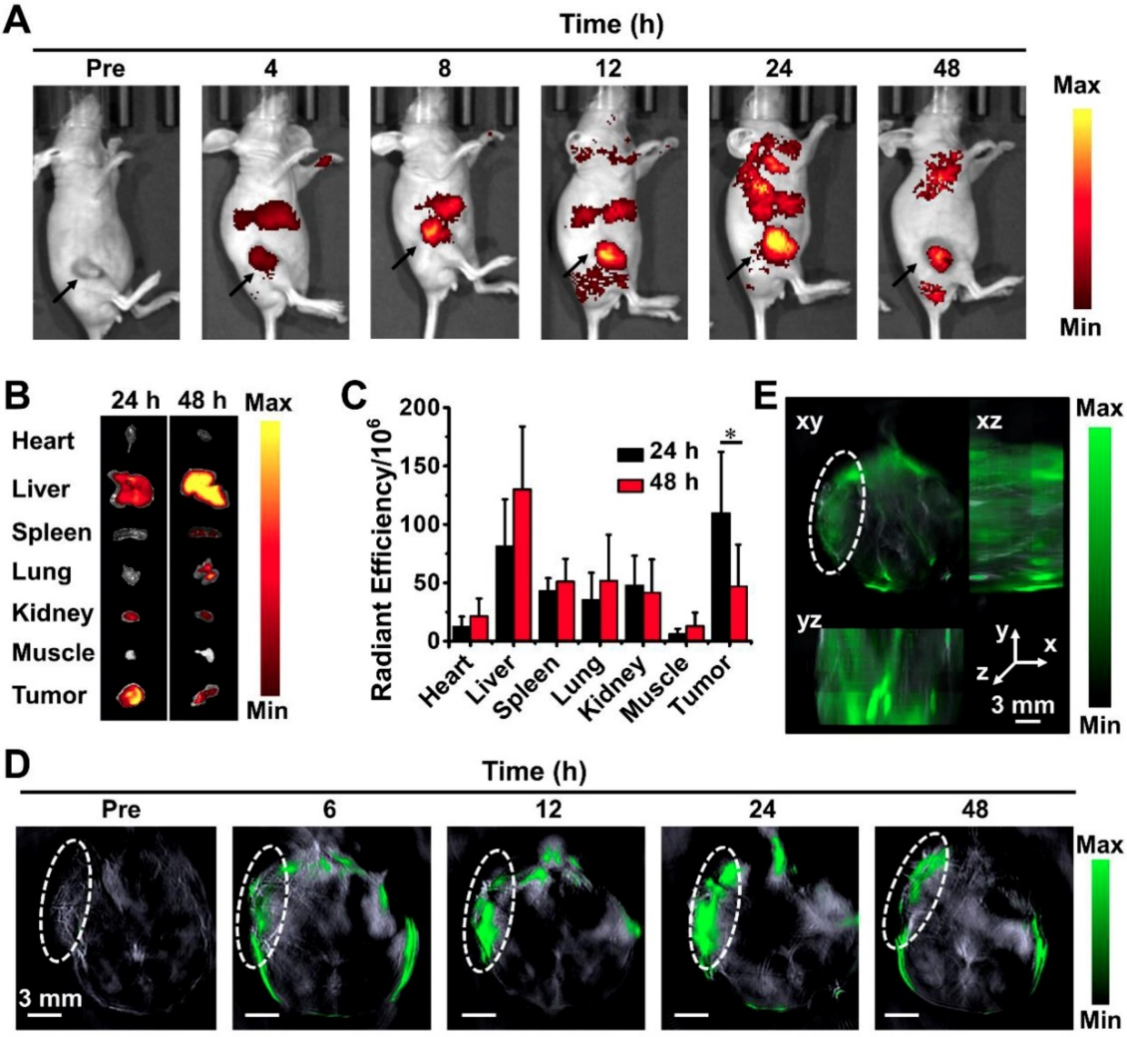
(A) *In vivo* NIRF imaging of the tumor-bearing mouse after intravenous injection of PPDI-NPs. (B) *Ex vivo* images and (C) the average radiant efficiencies (p·s^-1^·cm^-2^·sr^-1^)/ (mW·cm^-2^) of the major organ and tumors harvested at 24 h and 48 h post-injection. (D) Representative PA imaging of tumor-bearing mouse taken at different time points following PPDI-NPs injection. Scale bar = 3 mm. (E) 3D MSOT image at 24 h. Scale bars = 3 mm. The data were given as mean ± standard deviation (SD), *n* = 3, (*) *p* < 0.05.

Having confirmed the blood circulation behavior of PPDI-NPs, we proceeded to test their imaging ability in 4T1 tumor bearing mice *in vivo* by NIRF imaging and PA imaging. As shown in Figure 4A, a strong and sustained emission was observed, and the emission was gradually enhanced along with the increase of time, which is quantified in Figure S17A. Interestingly, the tumor tissue could be clearly distinguished from the surrounding normal tissues at 24 h with less tissue interference and exhibited high tumor-to-background contrast. The major organs were harvested and imaged by the IVIS system for *ex vivo* imaging at 24 h and 48 h, respectively. As shown in Figure 4B, the tumor showed a strong signal at 24 h post-injection. The quantitative analysis of fluorescence signal in organs confirmed that PPDI-NPs were effectively enriched at tumor at 24 h via the enhanced permeability and retention (EPR) effect (Figure 4C). PPDI-NPs have absorbance and emission in NIR region, where lights can efficiently pass through biological tissues (e.g. skin) than visible lights 57. Moreover, the emission could be enhanced in acidic microenvironment, resulting in a high contrast between tumor and normal organs and offering precise tumor detection.

Based on photoacoustic effect, MSOT imaging overcomes optical diffusion limitation by integrating the spectral selectivity of molecular excitation with high resolution of ultrasound detection. It is an advanced imaging technique with deep tissue penetration and fine spatial resolution. According to Figure 4D, PPDI-NPs' PA signal was detectable in body at 6 h after injected. Starting at 12 h post-injection, the nanoparticles were predominantly accumulated in the cancerous tissue. The PA signal indicated the maximal tumoral accumulation at 24 h post-injection with an 8-fold higher tumoral PA signal intensity than the basal level examined before injection (Figure S17B). Importantly, strong PA signal was found in the core of the tumor. Using continuous tomography, a 3D reconstruction of tumor at 24 h was performed to determine the precise spatial location of PPDI-NPs (Figure 4E). This result indicates that the suitable size (~60 nm) and biocompatible PEG shell contributed to the enrichment of PPDI-NPs through EPR effects. Over all, PPDI-NPs can act as efficient contrast agents for real-time NIRF and PA imaging.

### *In vivo* IRT imaging and PTT of PPDI-NPs

**Figure 5:**
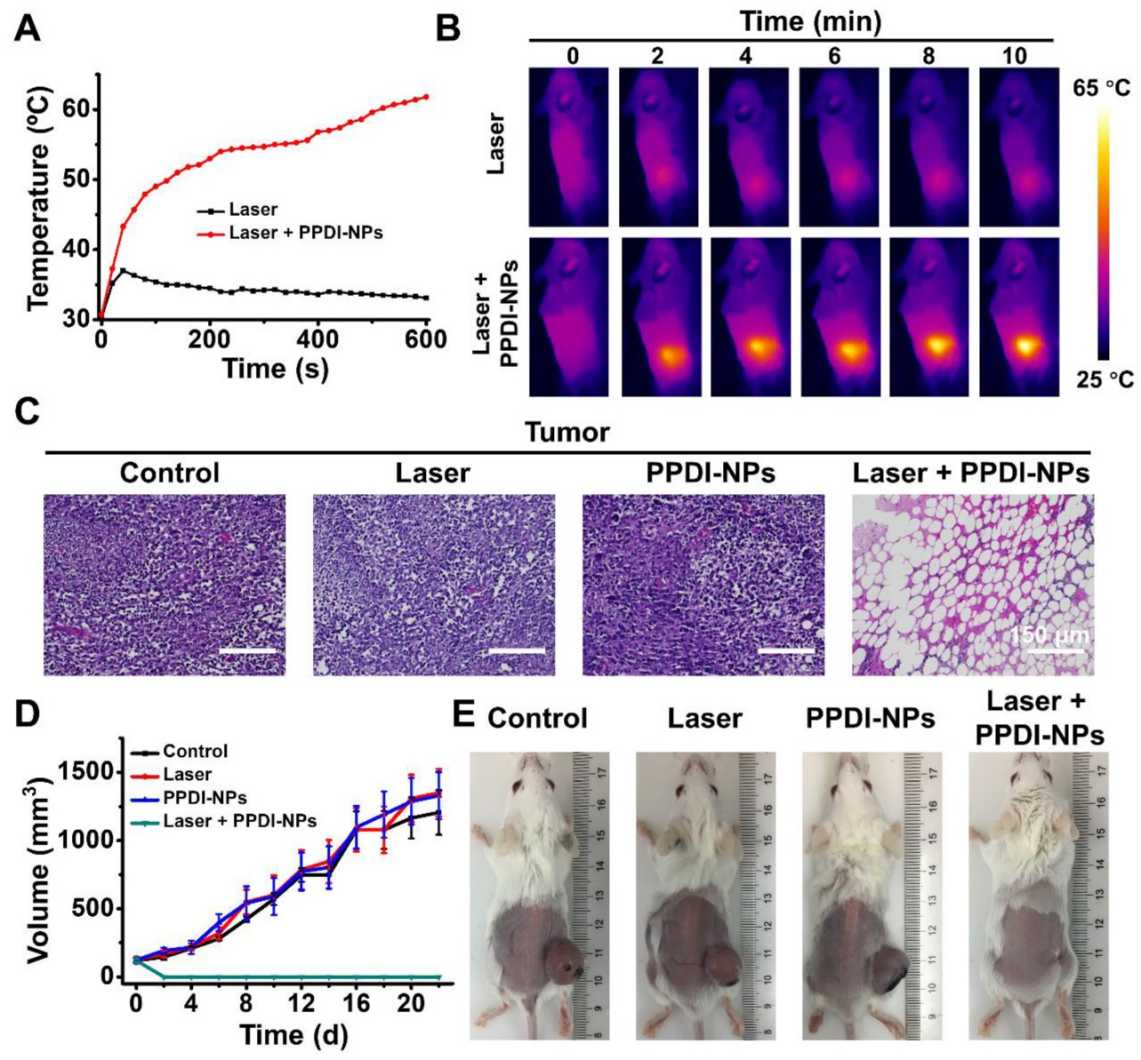
(A) Temperature elevations and (B) IRT imaging at the tumor sites exposed to laser (660 nm, 0.5 W/cm^2^, 10 min). (C) H&E stained tumor slices of different mice groups. Scale bars = 150 μm. (D) Tumor volume fluctuation (*n* = 3) and (E) digital photos of different treatment groups at the end of the experiment (22 d).

Encouraged by the outstanding NIRF/PA imaging and efficient accumulation of PPDI-NPs, we further investigated PPDI-NPs' IRT imaging and PTT abilities *in vivo*. When tumor size reached about 125 mm^3^, BALB/c mice were divided into four groups for various treatments: (1) PBS injection, (2) PPDI-NPs injection, (3) laser irradiation, and (4) PPDI-NPs injection with laser irradiation. The treated mice were i.v. injected with PPDI-NPs and the tumor sites were exposed to laser irradiation (660 nm laser, 0.5 W/cm^2^, 10 min) at 24 h post injection, as the “treatment-window” provided by NIRF/PA imaging. For mice with laser irradiation, the IRT imaging and temperature data were monitored by a thermal imaging camera. The mice groups after laser treatment showed sharply increased temperature by nearly 34 °C within 10 s and reached a very high temperature of 63 °C within 10 min (Figure 5A), which are highly favorable to thermally destroy tumor cells *in vivo*. In contrast, applying laser without PPDI-NPs did not cause significant temperature changes in the tumor of control group. As expected, real-time IRT imaging of the PPDI-NPs was observed (Figure 5B). Combining NIRF imaging and IRT imaging, the tumor location can be precisely identified (Figure S18A). The heat diffusion to the surrounding tissues was shown in Figure S18B. Importantly, the temperature change of the surrounding tissue was little compared with the tumor region, indicating less heat diffusion to surrounding tissue. The results confirmed that IRT imaging and PTT were selectively located at the target site (tumor). Subsequently, the PTT effect of tumor tissues was confirmed by histological examination (Figure 5C). The H&E stained tumor slices showed that PPDI-NPs caused sever tumor necrosis after laser irradiation, which is a typical sign of thermal cell necrosis damage 58.

After a single PTT process, the body weight and tumor volume of each group were recorded after 22 days in order to assess the therapy performances of PPDI-NPs. As shown in Figure 5D, tumors grew rapidly in the control groups and quickly festered. In contrast, the tumors of treated group were eliminated after PTT treatment. During the observational period, there were no abnormalities in the daily behavior of the mice in group (4), and the monitored body weight values were steady (Figure S19). 4T1 is a kind of malignant tumor that are easy to metastasize. The tumor can affect the normal physiological activity of mice, including food intake 59. The digital photos (Figure 5E) were taken in the at the end of the experiment (22 d), when the mice were thin in body with a large tumor except the group of Laser + PPDI-NPs. At the end of the experiment, the tumors of group (4) were disappeared with a small scare (Figure 5E). Moreover, the mice in groups (1) -(3) were gradually dead in 30 days of post-treatment, which probably were resulted from the malignant proliferation and/or abnormal lung metastasis of the tumor 60, 61. In contrast, the lifetime of the mice from group (4) can be substantially prolonged in 30 days of post-treatment. All the results indicated that PPDI-NPs have high PTT efficacy and can effectively inhibit the growth of tumor *in vivo*.

### *In vivo* toxicity assessment of PPDI-NPs

**Figure 6:**
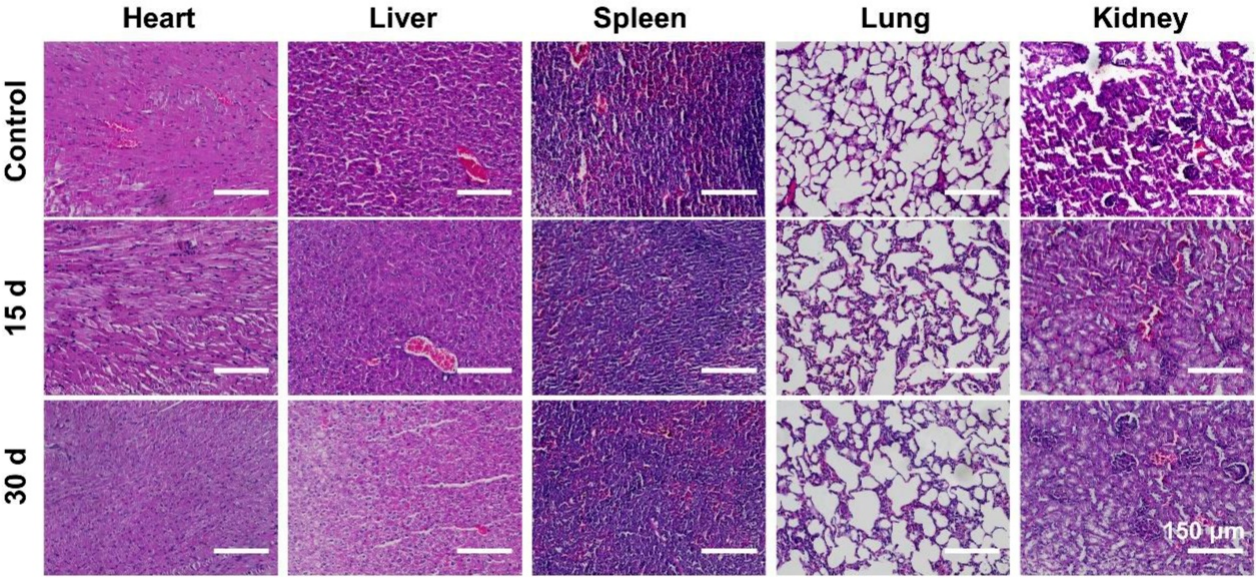
H&E images of major organs (heart, liver, spleen, lung, and kidney) collected from different time points. Scale bars = 150 μm.

Since the potential *in vivo* toxicity is a crucial consideration for practical applications 62, the behaviors of mice after being injected with the PPDI-NPs were carefully monitored. Healthy mice were divided into two groups (*n* = 3) and intravenously injected with PPDI-NPs. Mice were sacrificed at 15-day and 30-day post-injection, respectively. A group of untreated mice were used as control (*n* = 3). During the experiment, no obvious signs of toxic effects, such as abnormal eating, drinking, grooming, activity, exploratory behavior, urination, or neurological behavior were observed. To further assess the *in vivo* toxicity, a serum biochemistry assay was carried out. As shown in Figure S20, there is no significant difference in the concentration of measured parameters, including liver function markers (AST and ALT) and kidney function markers (CR, UA and BUN). Moreover, no noticeable tissue damage and adverse effects on major organs were found from the H&E stained organ slices as compared with the control group (Figure 6). PPDI-NPs had no significant side effects on mice during 30 days of short-term observations. The results indicated that PPDI-NPs were biocompatible for *in vivo* biomedical applications.

## Conclusion

In summary, we developed a pH-responsive perylenediimide as a multifunctional phototheranostic agent in a facile strategy. The perylenediimides was well dissolved in water to form stable nanoparticles (PPDI-NPs) with narrow size, which is suitable for high stability and long circulation. Importantly, the protonation of piperazine ring blocks the PET process in acidic microenvironment, leading to an increase of NIR emission of PDI at 760 nm. On the other hand, the “push-pull” effect can bring the photothermal property with PPDI-NPs. As a result, PPDI-NPs exhibited excellent NIR optical properties including fluorescence emission and photothermal effects. With the NIRF/PA/IRT trimodality imaging, the site and size of tumor could be accurately detected. Furthermore, high effective *in vivo* photothermal ablation of tumor had been achieved after the injection of PPDI-NPs and the irradiation of 660 nm laser, without appreciable toxicity. This study offered a new organic chromophore for developing phototheranostic cancer therapy.

## Supplementary Material

Supplementary figures.

## References

[1] Cheetham AG, Chakroun RW, Ma W, Cui H (2017). Self-assembling prodrugs. Chem Soc Rev.

[2] Cui H, Xu B (2017). Supramolecular medicine. Chem Soc Rev.

[3] Wang L, Yuan Y, Lin S, Huang J, Dai J, Jiang Q (2016). Photothermo-chemotherapy of cancer employing drug leakage-free gold nanoshells. Biomaterials.

[4] Wang Q, Tian L, Xu J, Xia B, Li J, Lu F (2018). Multifunctional supramolecular vesicles for combined photothermal/photodynamic/hypoxia-activated chemotherapy. Chem Commun (Camb).

[5] Gao D, Zhang B, Liu Y, Hu D, Sheng Z, Zhang X (2019). Molecular engineering of near-infrared light-responsive BODIPY-based nanoparticles with enhanced photothermal and photoacoustic efficiencies for cancer theranostics. Theranostics.

[6] Gao K, Tu W, Yu X, Ahmad F, Zhang X, Wu W (2019). W-doped TiO2 nanoparticles with strong absorption in the NIR-II window for photoacoustic/CT dual-modal imaging and synergistic thermoradiotherapy of tumors. Theranostics.

[7] Li W, Chen X (2015). Gold nanoparticles for photoacoustic imaging. Nanomedicine (Lond).

[8] Zheng T, Wang W, Wu F, Zhang M, Shen J, Sun Y (2019). Zwitterionic polymer-gated Au@TiO2 core-shell nanoparticles for imaging-guided combined cancer therapy. Theranostics.

[9] Yun SH, Kwok SJJ (2017). Light in diagnosis, therapy and surgery.

[10] Yang Z, Fan W, Tang W, Shen Z, Dai Y, Song J (2018). Near-infrared semiconducting polymer brush and pH/GSH-responsive polyoxometalate cluster hybrid platform for enhanced tumor-specific phototheranostics. Angew Chem Weinheim Bergstr Ger. 2018; 130: 14297-301; Angew Chem Int Ed Engl.

[11] Chen WH, Luo GF, Lei Q, Hong S, Qiu WX, Liu LH (2017). Overcoming the heat endurance of tumor cells by interfering with the anaerobic glycolysis metabolism for improved photothermal therapy. ACS Nano.

[12] Yang Z, Chen X (2019). Semiconducting perylene diimide nanostructure: multifunctional phototheranostic nanoplatform. Acc Chem Res.

[13] Yang Z, Song J, Tang W, Fan W, Dai Y, Shen Z (2019). Stimuli-responsive nanotheranostics for real-time monitoring drug release by photoacoustic imaging. Theranostics.

[14] Yang Z, Dai Y, Yin C, Fan Q, Zhang W, Song J (2018). Activatable semiconducting theranostics: simultaneous generation and ratiometric photoacoustic imaging of reactive oxygen species *in vivo*. Adv Mater.

[15] Dean-Ben XL, Gottschalk S, Mc Larney B, Shoham S, Razansky D (2017). Advanced optoacoustic methods for multiscale imaging of *in vivo* dynamics. Chem Soc Rev.

[16] Wang LV, Hu S (2012). Photoacoustic tomography: *in vivo* imaging from organelles to organs. Science.

[17] Hill TK, Kelkar SS, Wojtynek NE, Souchek JJ, Payne WM, Stumpf K (2016). Near infrared fluorescent nanoparticles derived from hyaluronic acid improve tumor contrast for image-guided surgery. Theranostics.

[18] Sun Y, Ma X, Cheng K, Wu B, Duan J, Chen H (2015). Strained cyclooctyne as a molecular platform for construction of multimodal imaging probes. Angew Chem Weinheim Bergstr Ger. 2015; 54: 5981-4; Angew Chem Int Ed Engl.

[19] Wang Q, Dai Y, Xu J, Cai J, Niu X, Zhang L (2019). All-in-one phototheranostics: single laser triggers NIR-II fluorescence/photoacoustic imaging guided photothermal/photodynamic/chemo combination therapy.

[20] Wang Q, Xia B, Xu J, Niu X, Cai J, Shen Q (2019). Biocompatible small organic molecule phototheranostics for NIR-II fluorescence/photoacoustic imaging and simultaneous photodynamic/photothermal combination therapy. Mater Chem Front.

[21] Liang X, Fang L, Li X, Zhang X, Wang F (2017). Activatable near infrared dye conjugated hyaluronic acid based nanoparticles as a targeted theranostic agent for enhanced fluorescence/CT/photoacoustic imaging guided photothermal therapy. Biomaterials.

[22] Wu Y, Gao D, Zhang P, Li C, Wan Q, Chen C (2016). Iron oxide nanoparticles protected by NIR-active multidentate-polymers as multifunctional nanoprobes for NIRF/PA/MR trimodal imaging. Nanoscale.

[23] Wang S, Lin J, Wang Z, Zhou Z, Bai R, Lu N (2017). Core-satellite polydopamine-gadolinium-metallofullerene nanotheranostics for multimodal imaging guided combination cancer therapy. Adv Mater.

[24] Ji C, Gao Q, Dong X, Yin W, Gu Z, Gan Z (2018). A size-reducible nanodrug with an aggregation-enhanced photodynamic effect for deep chemo-photodynamic therapy. Angew Chem Weinheim Bergstr Ger. 2018; 130: 11554-8; Angew Chem Int Ed Engl.

[25] Meng X, Yang Y, Zhou L, Zhang L, Lv Y, Li S (2017). Dual-responsive molecular probe for tumor targeted imaging and photodynamic therapy. Theranostics.

[26] Meng X, Zhang J, Sun Z, Zhou L, Deng G, Li S (2018). Hypoxia-triggered single molecule probe for high-contrast NIR II/PA tumor imaging and robust photothermal therapy. Theranostics.

[27] Hung CC, Huang WC, Lin YW, Yu TW, Chen HH, Lin SC (2016). Active tumor permeation and uptake of surface charge-switchable theranostic nanoparticles for imaging-guided photothermal/chemo combinatorial therapy. Theranostics.

[28] Zhang S, Guo W, Wei J, Li C, Liang XJ, Yin M (2017). Terrylenediimide-based intrinsic theranostic nanomedicines with high photothermal conversion efficiency for photoacoustic imaging-guided cancer therapy. ACS Nano.

[29] Li C, Zhang Y, Wang M, Zhang Y, Chen G, Li L (2014). *In vivo* real-time visualization of tissue blood flow and angiogenesis using Ag2S quantum dots in the NIR-II window. Biomaterials.

[30] Xiong H, Kos P, Yan Y, Zhou K, Miller JB, Elkassih S (2016). Activatable water-soluble probes enhance tumor imaging by responding to dysregulated pH and exhibiting high tumor-to-liver fluorescence emission contrast. Bioconjugate Chem.

[31] Uppal T, Bhupathiraju NVSDK, Vicente MGH (2013). Synthesis and cellular properties of Near-IR BODIPY-PEG and carbohydrate conjugates. Tetrahedron.

[32] Lu B, Chen Y, Li P, Wang B, Mullen K, Yin M (2019). Stable radical anions generated from a porous perylenediimide metal-organic framework for boosting near-infrared photothermal conversion. Nat Commun.

[33] Huth K, Glaeske M, Achazi K, Gordeev G, Kumar S, Arenal R (2018). Fluorescent polymer-single-walled carbon nanotube complexes with charged and noncharged dendronized perylene bisimides for bioimaging studies.

[34] Xu Z, Guo K, Yu J, Sun H, Tang J, Shen J (2014). A unique perylene-based DNA intercalator: localization in cell nuclei and inhibition of cancer cells and tumors. Small.

[35] Zheng Y, You S, Ji C, Yin M, Yang W, Shen J (2016). Development of an amino acid-functionalized fluorescent nanocarrier to deliver a toxin to kill insect pests. Adv Mater.

[36] Wang Q, Zhang P, Xu J, Xia B, Tian L, Chen J (2018). NIR-Absorbing dye functionalized supramolecular vesicles for chemo-photothermal synergistic therapy. ACS Appl Bio Mater.

[37] Ji C, Cheng W, Yuan Q, Müllen K, Yin M (2019). From Dyestuff chemistry to cancer theranostics: the rise of rylenecarboximides. Acc Chem Res.

[38] Chang S, Wu X, Li Y, Niu D, Gao Y, Ma Z (2013). A pH-responsive hybrid fluorescent nanoprober for real time cell labeling and endocytosis tracking. Biomaterials.

[39] Dubey RK, Knorr G, Westerveld N, Jager WF (2016). Fluorescent PET probes based on perylene-3,4,9,10-tetracarboxylic tetraesters. Org Biomol Chem.

[40] Aigner D, Borisov SM, Petritsch P, Klimant I (2013). Novel near infra-red fluorescent pH sensors based on 1-aminoperylene bisimides covalently grafted onto poly(acryloylmorpholine). Chem Commun (Camb).

[41] Bissell RA, Calle E, Silva APD, Silva SAD, Gunaratne HQN, Habib-Jiwan JL (1992). Cheminform abstract: luminescence and charge transfer. part 2. aminomethyl anthracene derivatives as fluorescent pet (photoinduced electron transfer) sensors for protons. J Chem Soc Perkin 1.

[42] Kraljić I, Mohsni SE (1978). A new method for the detection of singlet oxygen in aqueous solutions. Photochem Photobiol.

[43] Zou Q, Abbas M, Zhao L, Li S, Shen G, Yan X (2017). Biological photothermal nanodots based on self-assembly of peptide-porphyrin conjugates for antitumor therapy. J Am Chem Soc.

[44] Lyu Y, Xie C, Chechetka SA, Miyako E, Pu K (2016). Semiconducting polymer nanobioconjugates for targeted photothermal activation of neurons. J Am Chem Soc.

[45] Zhang J, Yang C, Zhang R, Chen R, Zhang Z, Zhang W (2017). Biocompatible D-A semiconducting polymer nanoparticle with light-harvesting unit for highly effective photoacoustic imaging guided photothermal therapy. Adv Funct Mater.

[46] Yuan A, Qiu X, Tang X, Liu W, Wu J, Hu Y (2015). Self-assembled PEG-IR-780-C13 micelle as a targeting, safe and highly-effective photothermal agent for *in vivo* imaging and cancer therapy. Biomaterials.

[47] Liu C, Zhang S, Li J, Wei J, Mullen K, Yin M (2019). A water-soluble, NIR-absorbing quaterrylenediimide chromophore for photoacoustic imaging and efficient photothermal cancer therapy. Angew Chem Weinheim Bergstr Ger. 2019; 131: 1652-6; Angew Chem Int Ed Engl.

[48] Zhang S, Li J, Wei J, Yin M (2018). Perylenediimide chromophore as an efficient photothermal agent for cancer therapy. Sci Bull (Beijing).

[49] Vincett PS, Voigt EM, Rieckhoff KE (1971). Phosphorescence and fluorescence of phthalocyanines. J Chem Phys.

[50] Tian Q, Hu J, Zhu Y, Zou R, Chen Z, Yang S (2013). Sub-10 nm Fe3O4@Cu(2-x)S core-shell nanoparticles for dual-modal imaging and photothermal therapy. J Am Chem Soc.

[51] Tang Q, Xiao W, Huang C, Si W, Shao J, Huang W (2017). pH-triggered and enhanced simultaneous photodynamic and photothermal therapy guided by photoacoustic and photothermal imaging. Chem Mater.

[52] Sun P, Wang X, Wang G, Deng W, Shen Q, Jiang R (2018). A perylene diimide zwitterionic polymer for photoacoustic imaging guided photothermal/photodynamic synergistic therapy with single near-infrared irradiation. J Mater Chem B.

[53] Sun P, Yuan P, Wang G, Deng W, Tian S, Wang C (2017). High density glycopolymers functionalized perylene diimide nanoparticles for tumor-targeted photoacoustic imaging and enhanced photothermal therapy. Biomacromolecules.

[54] Frackowiak D (1988). The jablonski diagram. J Photoch Photobio B.

[55] Ng KK, Zheng G (2015). Molecular interactions in organic nanoparticles for phototheranostic applications. Chem Rev.

[56] Song X, Zhang R, Liang C, Chen Q, Gong H, Liu Z (2015). Nano-assemblies of J-aggregates based on a NIR dye as a multifunctional drug carrier for combination cancer therapy. Biomaterials.

[57] Sordillo LA, Pu Y, Pratavieira S, Budansky Y, Alfano RR (2014). Deep optical imaging of tissue using the second and third near-infrared spectral windows. J Biomed Opt.

[58] Li Z, Liu J, Hu Y, Li Z, Fan X, Sun Y (2017). Biocompatible PEGylated bismuth nanocrystals: "all-in-one" theranostic agent with triple-modal imaging and efficient *in vivo* photothermal ablation of tumors. Biomaterials.

[59] Molanouri Shamsi M, Chekachak S, Soudi S, Quinn LS, Ranjbar K, Chenari J (2017). Combined effect of aerobic interval training and selenium nanoparticles on expression of IL-15 and IL-10/TNF-alpha ratio in skeletal muscle of 4T1 breast cancer mice with cachexia. Cytokine.

[60] Zhu H, Wang Y, Chen C, Ma M, Zeng J, Li S (2017). Monodisperse dual plasmonic Au@Cu2-xE (E= S, Se) core@shell supraparticles: aqueous fabrication, multimodal imaging, and tumor therapy at *in vivo* level. ACS Nano.

[61] Ovais M, Guo M, Chen C (2019). Tailoring nanomaterials for targeting tumor-associated macrophages. Adv.

[62] Guo Y, Wu Z, Shen S, Guo R, Wang J, Wang W (2018). Nanomedicines reveal how PBOV1 promotes hepatocellular carcinoma for effective gene therapy. Nat Commun.

