# Supplementary material for: pH-responsive perylenediimide nanoparticles for cancer trimodality imaging and photothermal therapy
Source: Theranostics. 2020 Jan 1;10(1):166–78. doi: 10.7150/thno.36999 (PMC6929613; doi:10.7150/thno.36999)
Supplement: Supplementary file 1 — Supplementary figures. [file thnov10p0166s1.pdf]

Supporting Information

pH-responsive perylenediimide nanoparticles for cancer trimodality imaging and photothermal therapy

Jianhao Li, Chang Liu, Yiseng Hu, Chendong Ji, Shuolin Li, and Meizhen Yin**

State Key Laboratory of Chemical Resource Engineering, Beijing Advanced Innovation Center for Soft Matter Science and Engineering, Beijing Laboratory of Biomedical Materials, Beijing University of Chemical Technology, No. 15 the North Third Ring Road East, Chaoyang District, Beijing 100029, PR China

Correspondence to M. Yin, Email: yinmz@mail.buct.edu.cn; C. Ji, Email: jicd@mail.buct.edu.cn

Supplementary Date

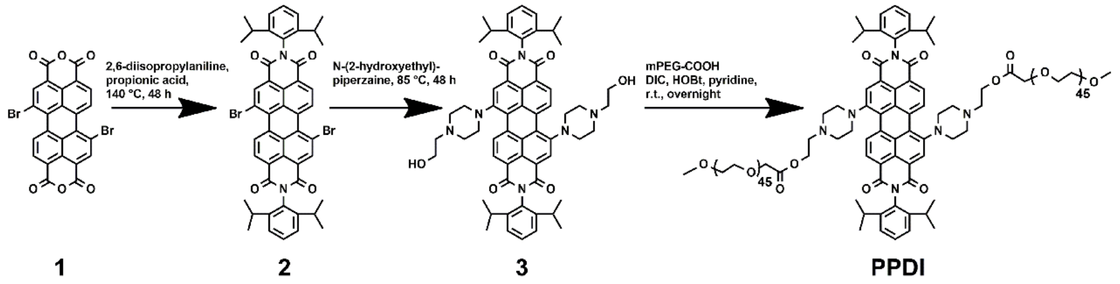

Scheme S1. Synthesis route of the **PPDI**.

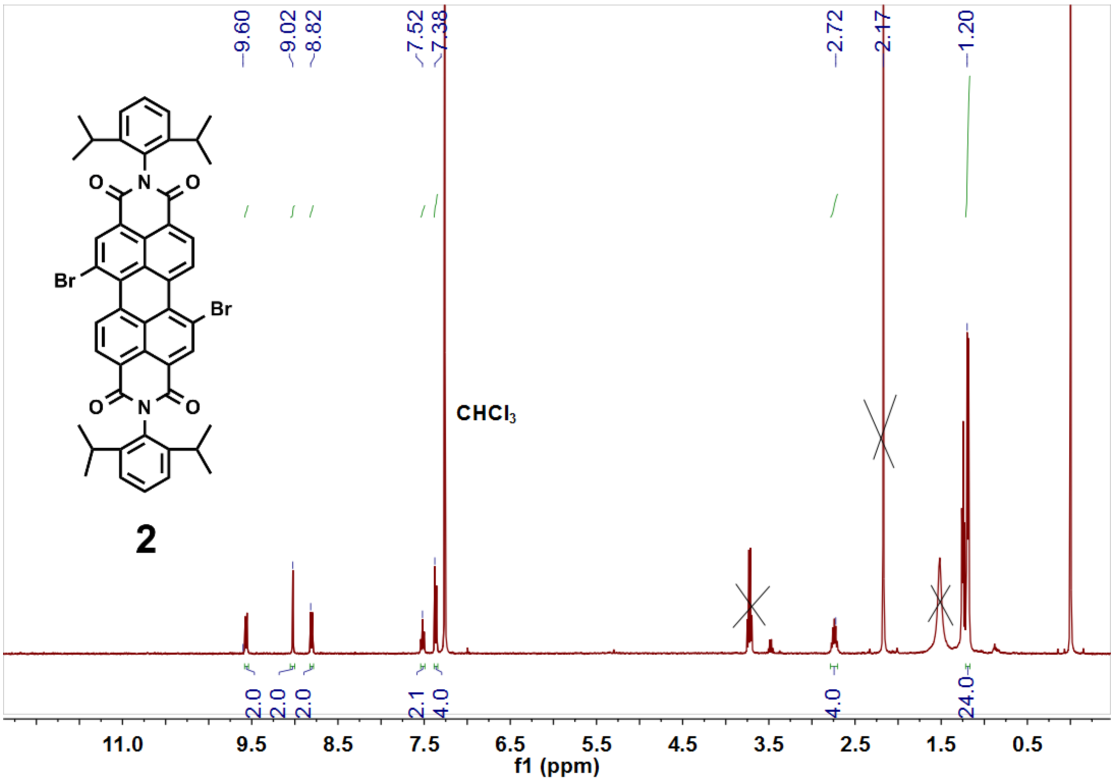

Figure S1. ^{1}H NMR spectrum of compound **2** in $CDCl_3$.

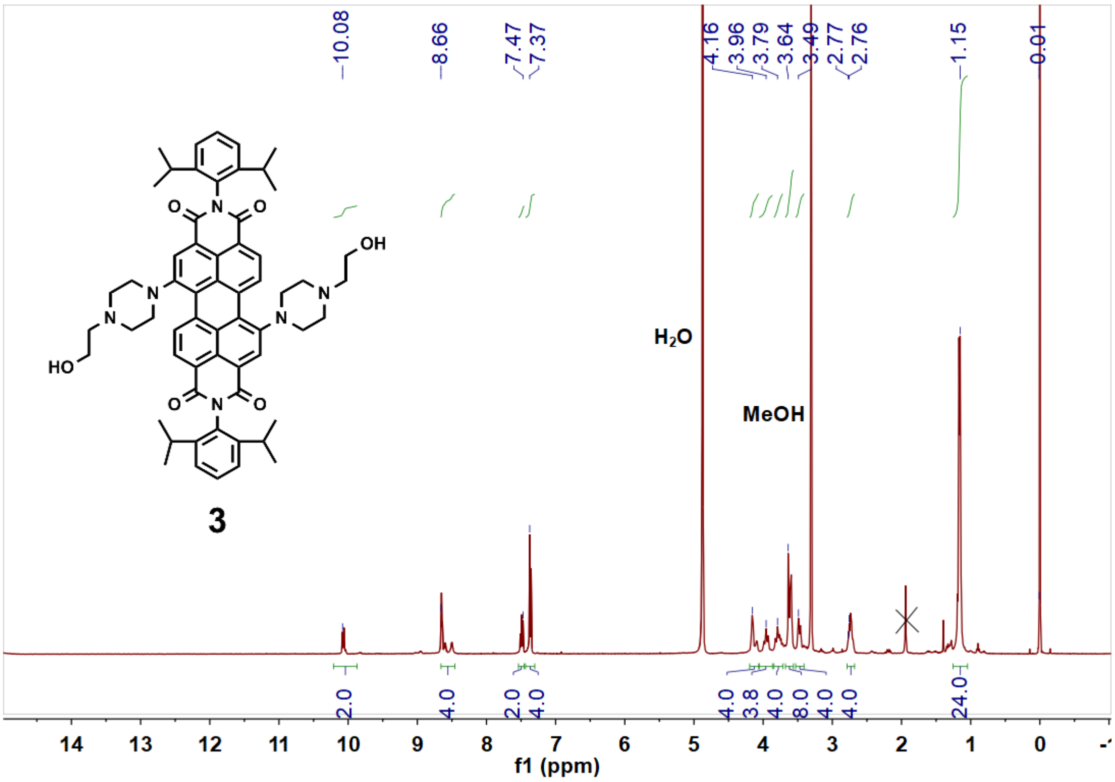

Figure S2. ^{1}H NMR spectrum of compound **3** in MeOD.

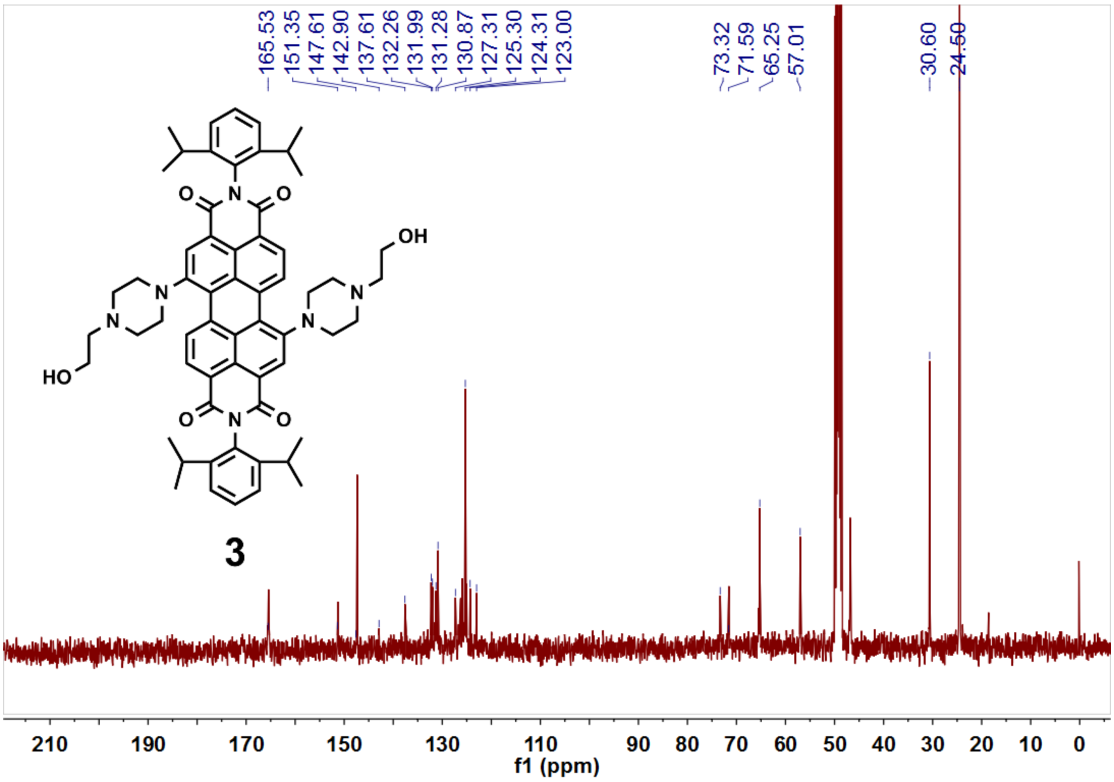

Figure S3. ^{13}C NMR spectrum of compound **3** in MeOD.

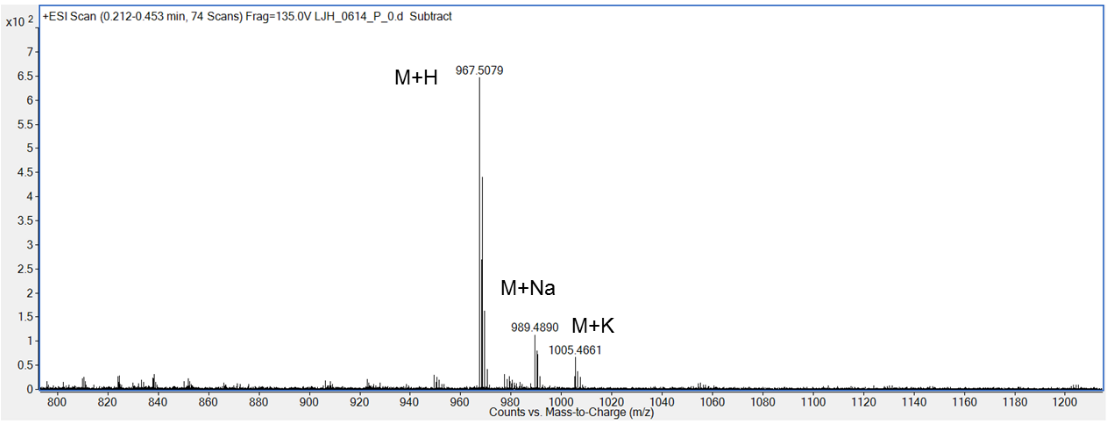

Figure S4. MS spectrum of compound **3**.

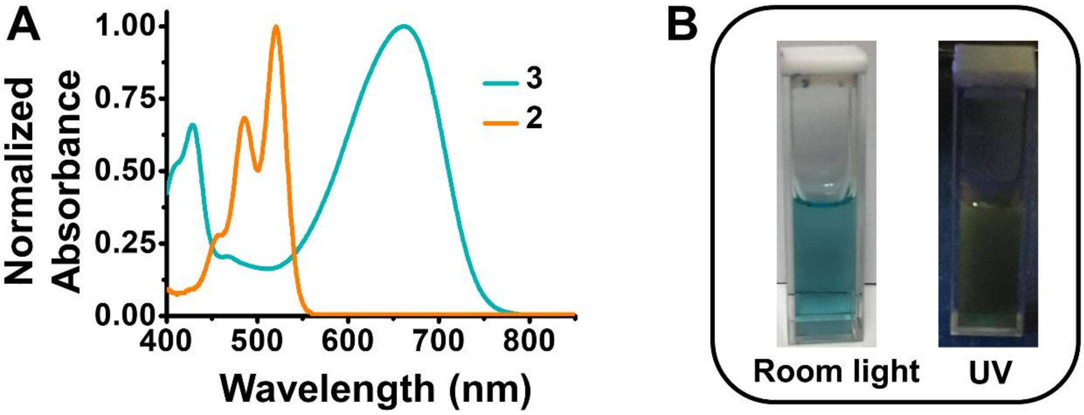

Figure S5. Optical properties of compound **3** in CH_2Cl_2. (A) Absorption spectrum. (B) Digital photographs under room light and UV light.

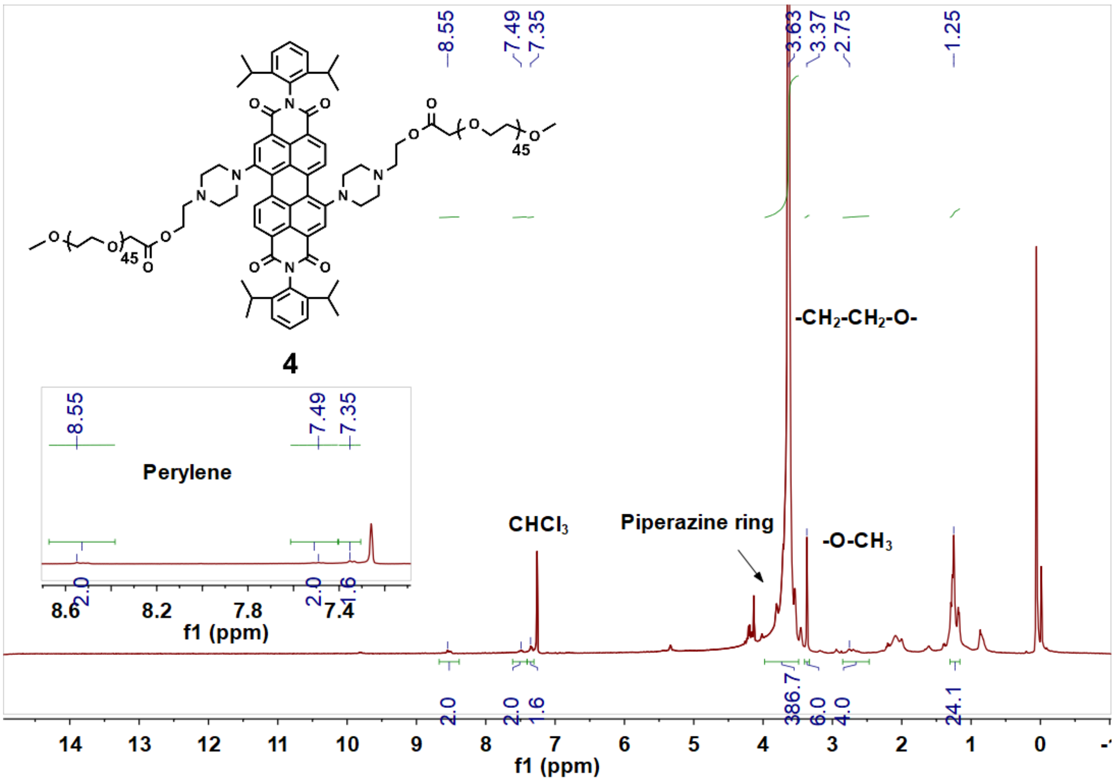

Figure S6. ^{1}H NMR spectrum of compound **PPDI** in $CDCl_3$.

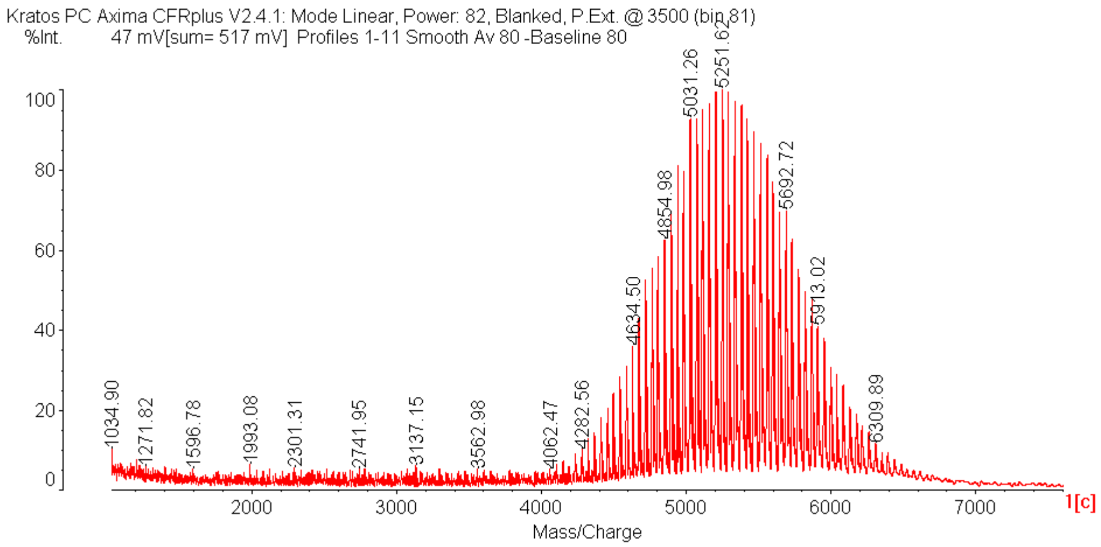

Figure S7. MAIDI-TOF-MS spectrum of compound **PPDI**.

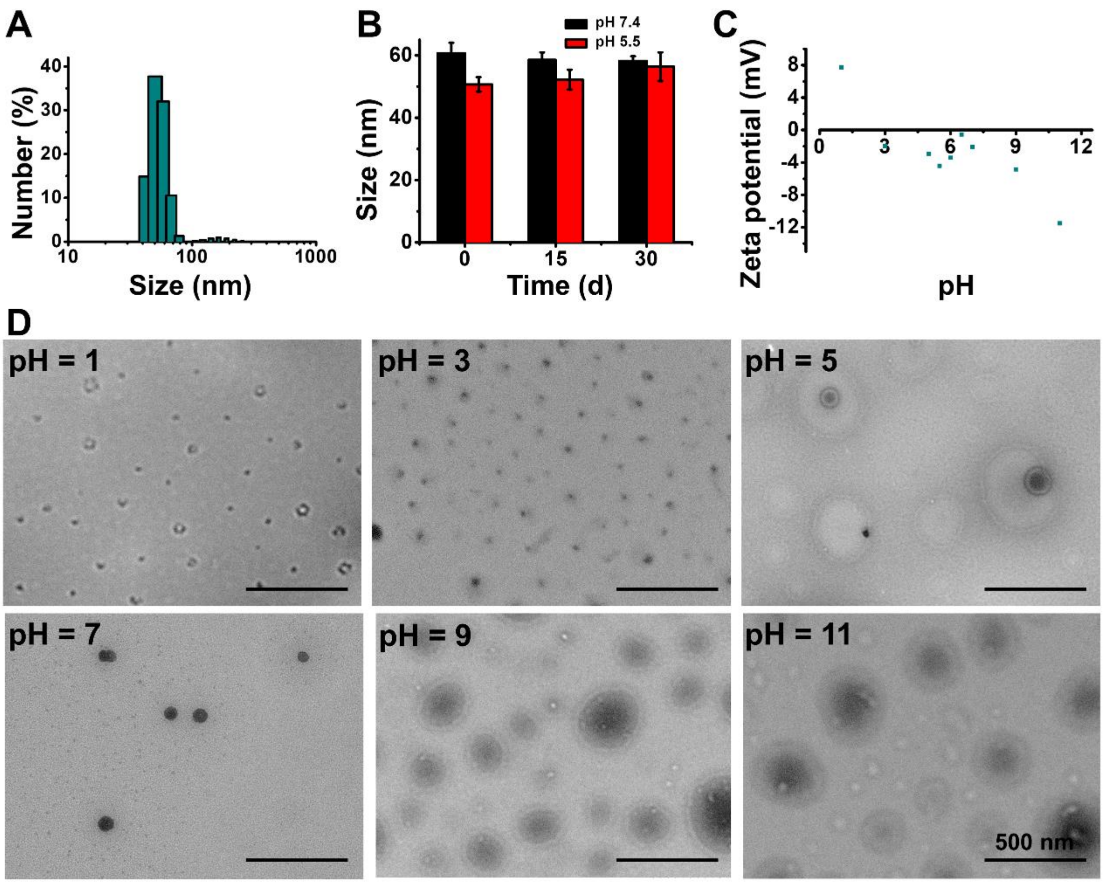

Figure S8. (A) DLS of PPDI-NPs in PBS (pH = 5.5). (A) DLS of PPDI-NPs in PBS for different days. (C) Zeta potential of PPDI-NPs at different pH. (D) TEM images of PPDI-NPs at different pH.

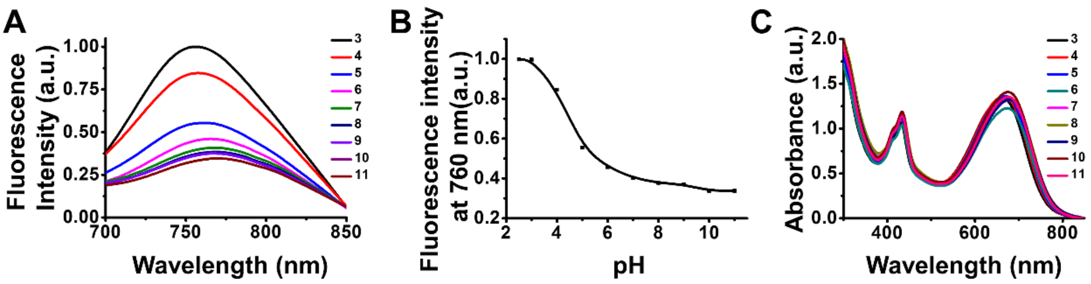

Figure S9. Optical properties of PPDI-NPs in PBS at different pH (A) Fluorescence spectrum. (B) Fluorescence intensity at 760 nm as the function of pH. (C) Absorbance spectrum.

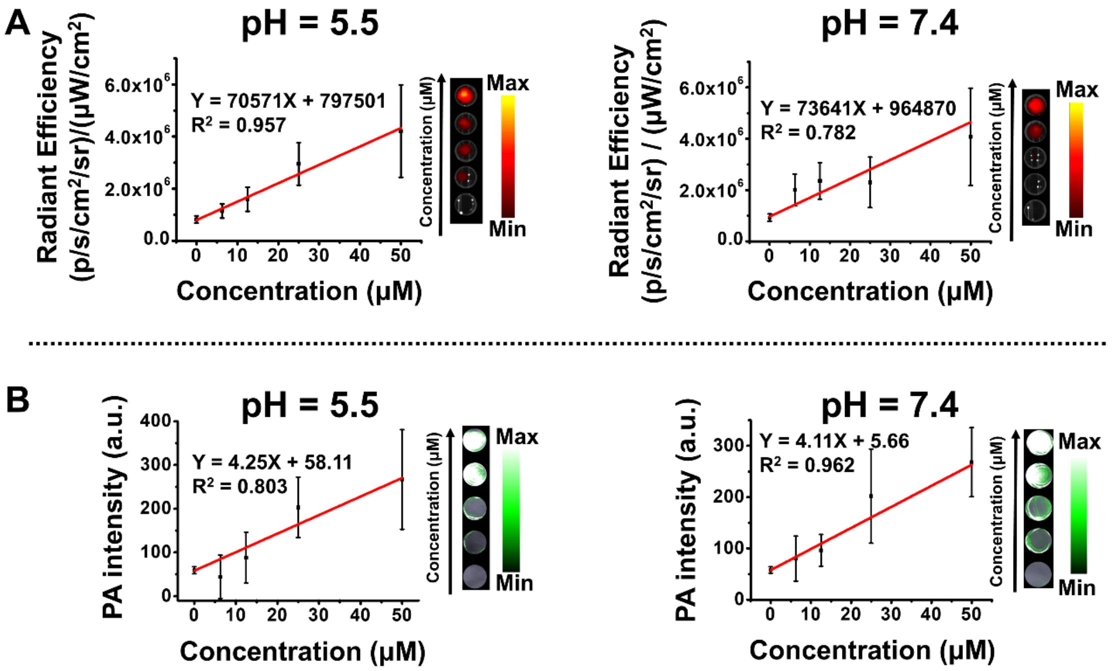

Figure S10. Concentration-dependent (A) NIRF imaging and (B) PA imaging of PPDI-NPs.

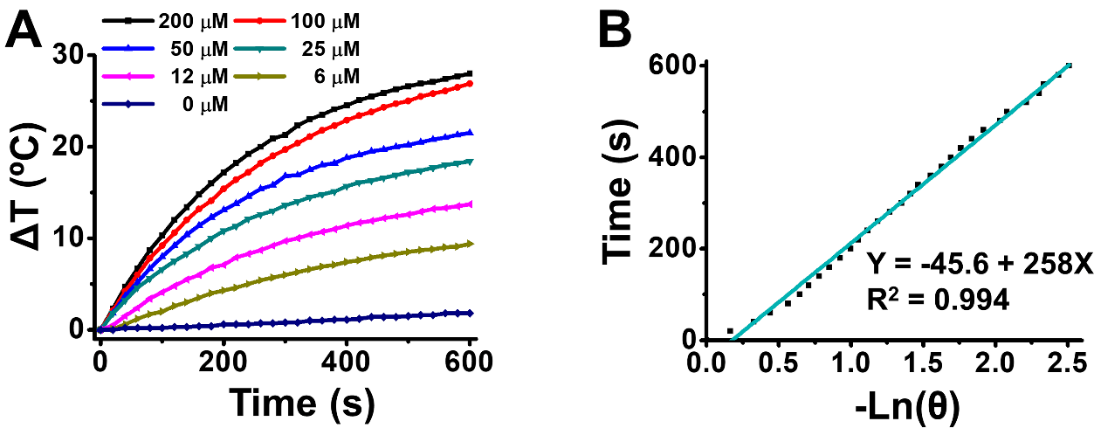

Figure S11. (A) Concentration-dependent temperature changes of PPDI-NPs (660 nm, 0.5 W/cm^2, 10 min). (B) Liner time data versus – *lnθ* obtained from the cooling period of Figure 2C.

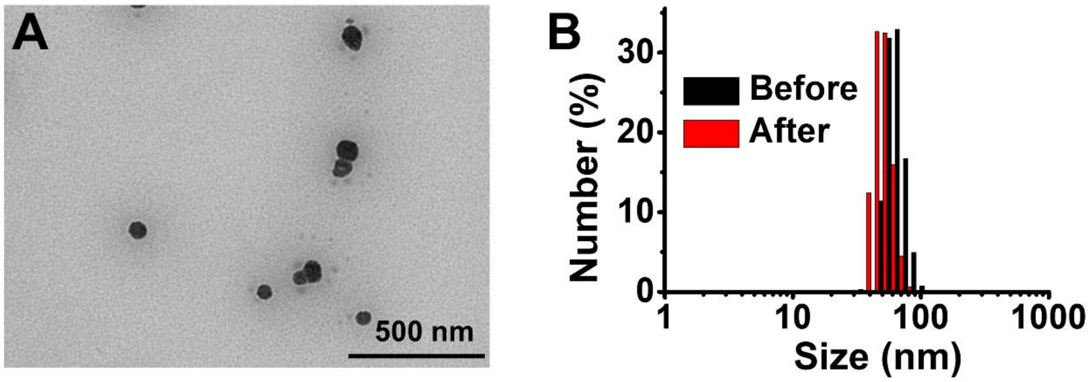

Figure S12. (A) TEM image of PPDI-NPs after laser irradiation. (B) DLS of PPDI-NPs before and after laser irradiation. The pdi value was 0.27 (before) and 0.34 (after), respectively (660 nm, 1 W/cm^2, 10 min).

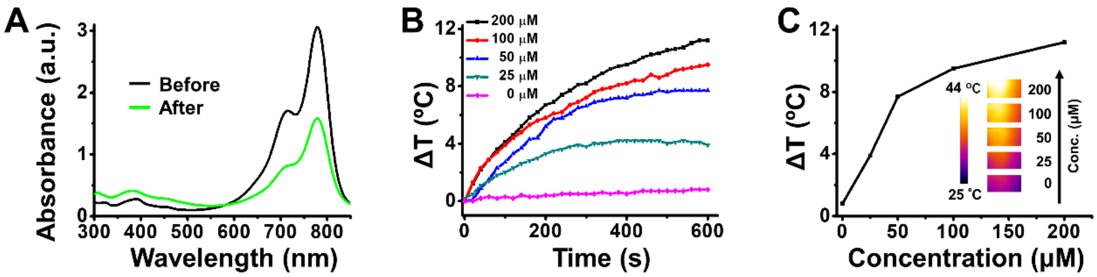

Figure S13. (A) Absorbance spectrum of ICG (100 μM) before and after laser irradiation (660 nm, 1 W/cm^2, 10 min). (B) Concentration-dependent temperature changes and (C) Plot of temperature change over a period of 10 min versus the concentration of ICG in aqueous solution. Inset picture is the infrared thermal images of ICG in aqueous solution in a cuvette under laser irradiation (660 nm, 0.5 W/cm^2, 10 min)

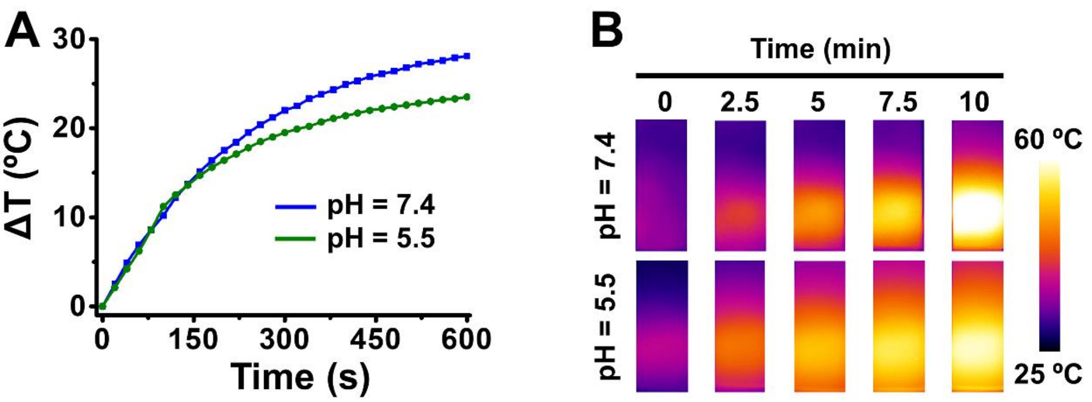

Figure S14. (A) Temperature change and (B) infrared thermal images of PPDI-NPs (100 μM) in PBS (pH = 5.5 and 7.4) exposed to laser irradiation (660 nm, 0.5 W/cm^2, 10 min).

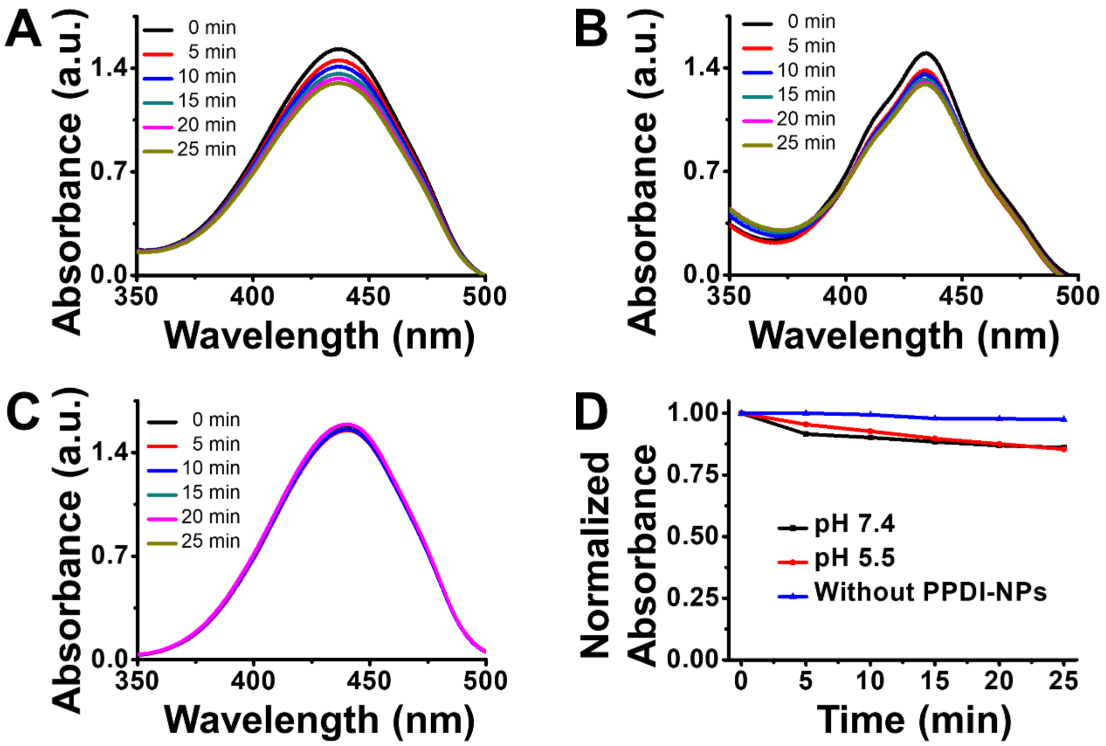

Figure S15. Absorbance spectrum of RNO solution with the presence of (A) PPDI-NPs at pH 5.5, (B) PPDI-NPs in pH 7.4 and (C)without PPDI-NPs under irradiation. (D) Plot showing the change in absorbance of RNO solution at 440 nm during 25 min of laser exposure (laser irradiation: 660 nm, 0.5 W/cm^2).

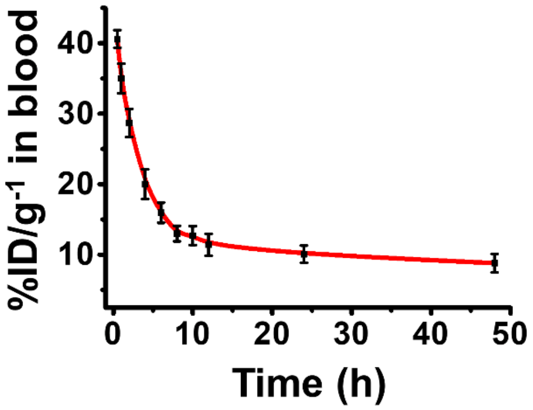

Figure S16. Blood circulation curves of PPDI-NPs determined by measuring the fluorescence intensity of PPDI-NPs in the blood at different time points after injection. The unit was a percentage of injected dose per gram tissue (% ID/g). Error bars were based on triplicated samples.

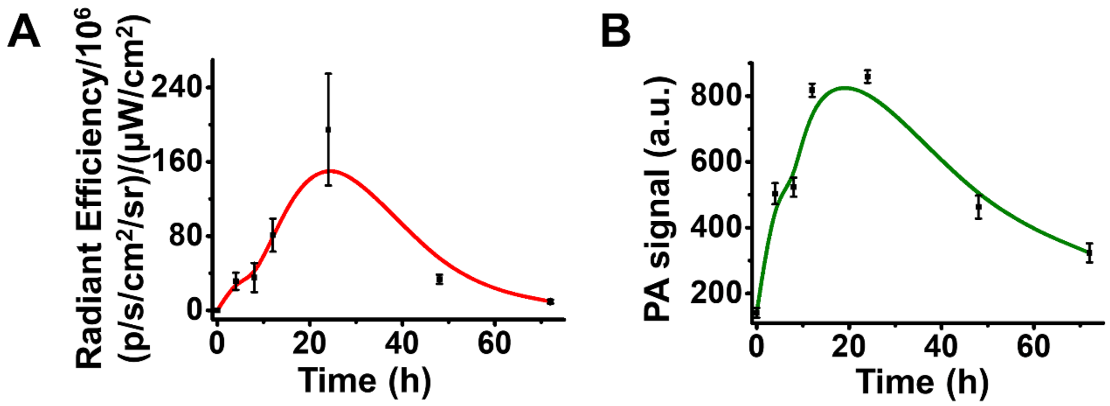

Figure S17. (A) NIRF intensity and (B) Quantification PA signal in tumor site at different time intervals. Error bars were based on triplicated samples.

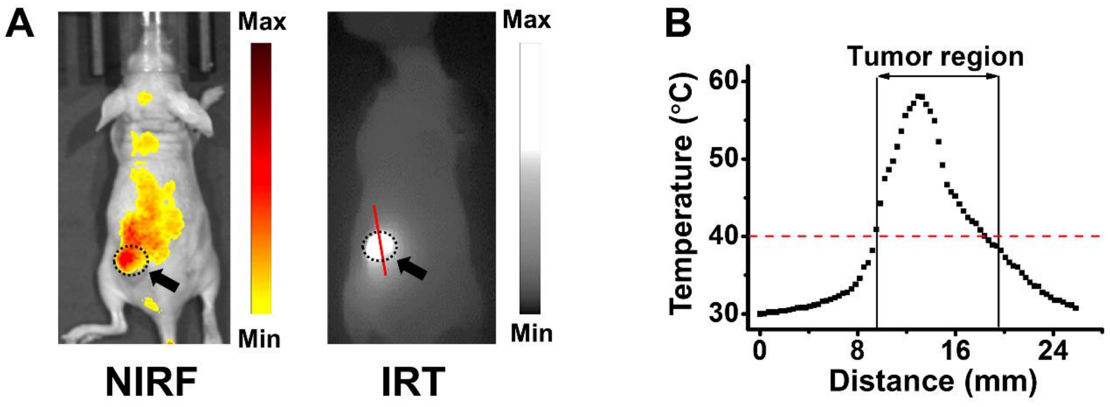

Figure S18. (A) NIRF images and IRT images of the tumor-bearing nude mice after intravenous injection of PPDI-NPs. The arrow shows the location of tumor (660, 0.5 W/cm^2,10 min). (B) Heat diffusion of the tissue section labeled in red line of Figure S18A.

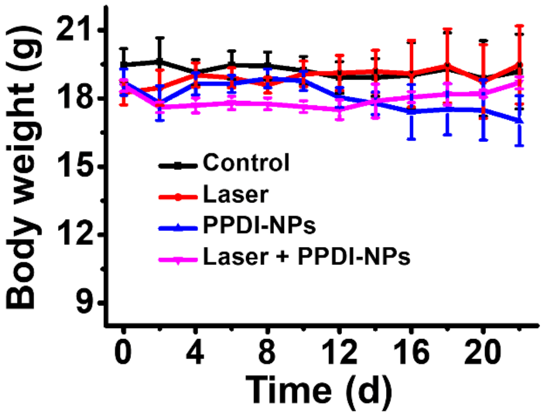

Figure S19. Body weights of mice (n = 3) at different time points after various treatments.

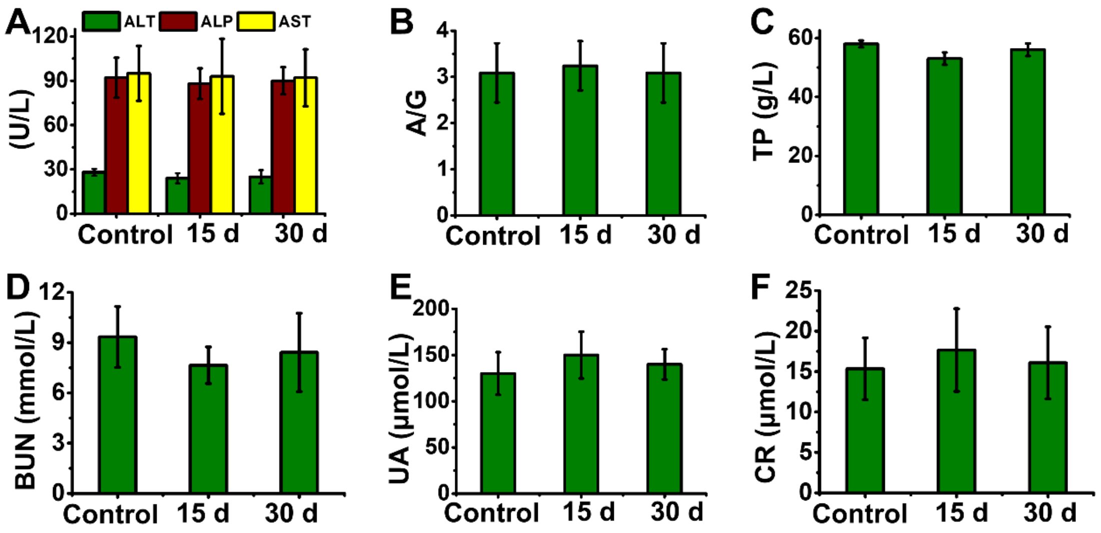

Figure S20. Blood biochemistry analysis of the mice treated with the PPDI-NPs at the different time. The results show mean and standard deviation of liver function markers (alanine aminotransferase (ALT), alkaline phosphatase (ALP) and aspartate aminotransferase (AST)), albumin/globin ratios (A/G), renal function markers (blood urea nitrogen (BUN), uric acid (UA) and creatinine (CREA)) and total protein (TP).
